# Generating multi-pathological and multi-modal images and labels for brain MRI

**DOI:** 10.1016/j.media.2024.103278

**Published:** 2024-10

**Authors:** Virginia Fernandez, Walter Hugo Lopez Pinaya, Pedro Borges, Mark S. Graham, Petru-Daniel Tudosiu, Tom Vercauteren, M. Jorge Cardoso

**Affiliations:** Department of Biomedical Engineering and Imaging Sciences, King’s College London, Strand, London, WC2R 2LS, United Kingdom

**Keywords:** 92C55, 68T45, 68T01, Generative modelling, Medical imaging segmentation, Brain MRI

## Abstract

The last few years have seen a boom in using generative models to augment real datasets, as synthetic data can effectively model real data distributions and provide privacy-preserving, shareable datasets that can be used to train deep learning models. However, most of these methods are 2D and provide synthetic datasets that come, at most, with categorical annotations. The generation of paired images and segmentation samples that can be used in downstream, supervised segmentation tasks remains fairly uncharted territory. This work proposes a two-stage generative model capable of producing 2D and 3D semantic label maps and corresponding multi-modal images. We use a latent diffusion model for label synthesis and a VAE-GAN for semantic image synthesis. Synthetic datasets provided by this model are shown to work in a wide variety of segmentation tasks, supporting small, real datasets or fully replacing them while maintaining good performance. We also demonstrate its ability to improve downstream performance on out-of-distribution data.

## Highlights

• 2D and 3D paired labels and multi-modal images are synthesised end-to-end.
• Conditioning enables synthesis of lesions and extrapolation to unseen combinations.
• Synthetic datasets can be used to supplement small real datasets for segmentation.
• Synthetic datasets improve generalisability to other domains and unseen lesions.
• Privacy analysis shows no memorisation of training datasets.

## 1 Introduction

Medical imaging segmentation involves the delineation of one or multiple regions of interest (ROIs) in a medical image via the assignation of an ROI class to relevant voxels in the image. The task, which requires radiological expertise, is traditionally performed manually and is not only time-consuming but can also give rise to inter- and intra-observer variability (Renard et al., 2020). Recent studies have shown that automated segmentation algorithms using Convolutional Neural Networks (CNN) can be a good surrogate for manual segmentation (Isensee et al., 2021), as they are fast and effective (Goodfellow et al., 2015).

### 1.1 Limitations with deep learning for image segmentation

Nonetheless, the application of CNN-based methods clinically encounters a significant limitation. If trained with scarce data, they can lead to overfitting on the training dataset, making them biased and ineffective towards unseen distributions (Goodfellow et al., 2015, Kaissis et al., 2020). Whereas large and comprehensive datasets are available in computer vision, this is not the case in medical imaging.

First, medical images are not as easy to acquire, all medical imaging modalities require costly equipment that needs expert manipulation. Secondly, medical images are Protected Health Information (PHI) and are subject to strict data protection policies (ESR, 2017), which results in most imaging datasets remaining private, without the possibility of being shared across institutions. Thirdly, available public datasets usually comply with a specific clinical criteria, excluding subjects who do not fulfil certain criteria, resulting in biases (Ricci Lara et al., 2022). Lastly, because most state-of-the-art segmentation methods are supervised (Anwar et al., 2018), manually drawn segmentations are required for training. As stated above, these are hard to obtain, typically resulting in available segmentations being constrained to a single task, often resulting in overly specific, small, and biased datasets.

### 1.2 What approaches can we use to overcome the shortfall of medical imaging datasets?

One of the approaches is to make the segmentation models robust to incomplete, small or biased datasets. Approaches using multi-task learning to tackle incomplete datasets (Dorent et al., 2021), domain adaptation (Guan and Liu, 2022) or randomisation (Billot et al., 2023) can be used to improve gaps between data distributions. However, most of these approaches tend to produce models tailored to specific tasks. A more generic strategy is to use deep learning-based data augmentation, which increases the size of training datasets along relevant directions of variability (Chlap et al., 2021). Traditional data augmentation is defined by the user and typically consists of an aggregated and randomised set of simple mathematical transformations on the images. Still, it does not necessarily model real variations in the input data distributions and is limited by the user’s knowledge about the latter.

Generative modelling is a class of unsupervised learning algorithms that models an input data distribution and does not require the user to have prior knowledge about its characteristics (Goodfellow et al., 2015). Most state-of-the-art generative models are deep learning-based, such as generative adversarial networks (GANs), first introduced in Goodfellow et al. (2014), variational auto-encoders (VAEs) (Kingma and Welling, 2014), or the state-of-the-art diffusion models (Rombach et al., 2021). One main advantage of using deep learning-based generative models is that their latent representation of the data distribution typically allows for stochastic sampling, resulting in a source of infinite synthetic data. However, most of the state-of-the-art studies in medical imaging focus on only generating images, which makes them unsuitable for training supervised methods such as state-of-the-art nnU-Net (Isensee et al., 2021), due to the absence of corresponding labels also being generated.

In magnetic resonance imaging (MRI) of the brain, where acquired contrasts are chosen to highlight the specific physiological and/or pathological traits required by the study being conducted, there is an argument to be made in favour of generative models that are capable of providing physiological and pathological multi-modal images and associated labels, as they will have the potential to augment datasets for a wide range of brain segmentation tasks.

## 2 Related works

Recent works in the field of computer vision have shown that synthetic data generated using a generative model can improve the performance of downstream classification tasks (Azizi et al., 2023), including in the medical imaging domain (Motamed et al., 2021, Khader et al., 2023). Using different base architectures, including VAEs, GANs, Vector-Quantised VAEs (van den Oord et al., 2017) or the state-of-the-art diffusion models (Ho et al., 2020), numerous works have been published on the topic of data augmentation via generative modelling for medical imaging (Kazerouni et al., 2023), even leading to open-source libraries and databases such as MEDIGAN (Osuala et al., 2022) or ‘MONAI Generative Models’ (Pinaya et al., 2023).

Despite this notable progress, some challenges remain, especially when generating brain MRI images for segmentation. First, brain MRI images are volumetric, unlike most natural images used in computer vision. State-of-the-art brain segmentation algorithms are 3D (Isensee et al., 2021), but the availability of 3D generative models is limited due to the large computational resources required to train them. To this date, relatively few examples in the literature have deployed 3D generative models for brain MRI, such as Tudosiu et al., 2022, Sun et al., 2022, Khader et al., 2023 or Pinaya et al. (2022), even though these examples remain constrained to healthy data or single pathologies.

The second challenge is that of conditioning. Many published works do not offer the user any tool to constrain the nature of generated images, leading to models limited to a modality or pathological profile (Sun et al., 2022). The publication of text encoders and their coupling to diffusion models has enabled high-quality text-based conditioning (Chambon et al., 2022), which has proven useful for X-ray imaging. Diffusion models have also been coupled to age or ventricular size conditioning (Pinaya et al., 2022). More appealing in the context of using synthetic images for segmentation is the existence of semantic synthesis models, such as modified diffusion models which accept masks (Dorjsembe et al., 2023), or SPADE normalisation (Park et al., 2019), that allows for conditioning on a semantic map. SPADE has been applied in various medical imaging 2D applications (Qasim et al., 2020, Fernandez et al., 2022, Stojanovski et al., 2023), resulting in synthetic images paired to segmentations that can be used to supplement or replace real datasets during the training of supervised segmentation algorithms. These methods are, nonetheless, limited to 2D.

One additional problem is the source of labels. Brain segmentation models are usually tailored to the dataset task (e.g. tumour segmentation) and rarely comprehend physiological and multi-pathological information. This narrow scope limits the applicability of SPADE-based methods, which require a rich semantic coverage of the images. In our previous work (Fernandez et al., 2022), we developed a generative model, brainSPADE, for paired 2D multi-modal brain MRI images and corresponding labels, by coupling a diffusion model-based label generator to a modified SPADE network. In that work, we showed the potential of applying healthy and diseased synthetic images to segmentation tasks, improving segmentation performance when real datasets are small, or addressing out-of-distribution issues. brainSPADE was, however, restricted to 2D images; and a different label generator had to be trained to allow for labels with specific pathological profiles.

## 3 Contributions

In this work, we extend brainSPADE (Fernandez et al., 2022) by making the following additions:

• In addition to re-implementing the 2D approach, with 2D label and image generators, we extend our method to 3D by implementing a 3D label generator, resulting in 3D labels that can be forwarded, slice by slice, to the 2D image generator, then assembled into volumetric images.^1^ We minimise the impact of generating each image slice independently by incorporating losses that increase the consistency of the contrast of images corresponding to different slices, and run an ablation study on these losses and additions.
• We implement conditioning mechanisms in the 2D and 3D label generators to allow for control over which pathologies are present within the synthetic labels.
• Apply generated 2D and 3D datasets to a wide range of segmentation tasks, including out-of-distribution segmentation and pathology segmentation, showing that synthetic data from our model can be used to train segmentation networks that generalise well to real data.

## 4 Materials and methods

### 4.1 Data

To incorporate multiple pathologies and contrasts into our model training, we used a wide range of brain MRI datasets to train the label and image generators and to run the validation experiments. Dataset sizes were chosen to test for model resilience to scales of data:

• **Southall and Brent Revisited V3 (SABRE v3)** (Jones et al., 2020).
• **Alzheimer’s Disease Neuroimaging Initiative 2 (ADNI 2)**^2^ We used an internal pre-processed subset.
• **Brain Segmentation Tumour Challenge (BraTS) 2020**: we split BraTS into subjects from the Cancer Imaging Archive (“BraTS-TCIA”) site and the rest of the sites (“BraTS-OTHER”).
• **Open Access Series of Imaging Studies (OASIS)** (LaMontagne et al., 2019).
• **Autism Brain Imaging Data Exchange (ABIDE)** (Di Martino et al., 2014): we used the subset of subjects from the Yale site.

These datasets were registered via an affine transform to the ICBM T1 1 mm isotropic template using ANTsPy (https://github.com/ANTsX/ANTsPy), resulting in $\left(193\times 229\times 193,1\;{\mathrm{mm}}^{3}\right)$ volumes. T1 images were used for the registration, and the resulting transform matrix was applied to the other modalities and segmentations.

Table 1 summarises the characteristics of the datasets used in our experiments. We used GIF (Cardoso et al., 2012) to obtain generated probabilistic labels for cerebrospinal fluid (CSF), grey matter (GM), white matter (WM), deep grey matter (DGM) and brainstem. Lesion segmentations were provided with the datasets and fused with the above-mentioned healthy tissue maps. Each healthy tissue being in a different channel, we concatenated the lesion masks on additional channels, setting the values of healthy tissues to zero for voxels where the lesion was 1.

The 2D models we used were trained on slices taken from these 3D datasets. The slicing process is detailed in the appendix (Appendix B.1).

**Table 1:** Summary of data used for this work. Abbreviations: LG: label generator, IG: image generator, EXP: segmentation experiments; GD-E: gadolinium-enhanced; NE: non-enhanced tumour.

Dataset	Split	n^o^ subjects	Modalities	Pathologies	Used in...
SABRE	Train	583	T1, FLAIR, T2	WMH	LG, IG, EXP
	Test	54	T1, FLAIR, T2	WMH	EXP

ADNI-2	–	98		WMH	
ABIDE	–	29		–	
OASIS	–	71		WMH	

BraTS		129	T1, FLAIR, T2	NE tumour, GD-E tumour and oedema	LG, IG, EXP
		31	T1, FLAIR, T2	NE tumour, GD-E tumour and oedema	EXP

### 4.2 Model and training pipeline

**Fig. 1:**
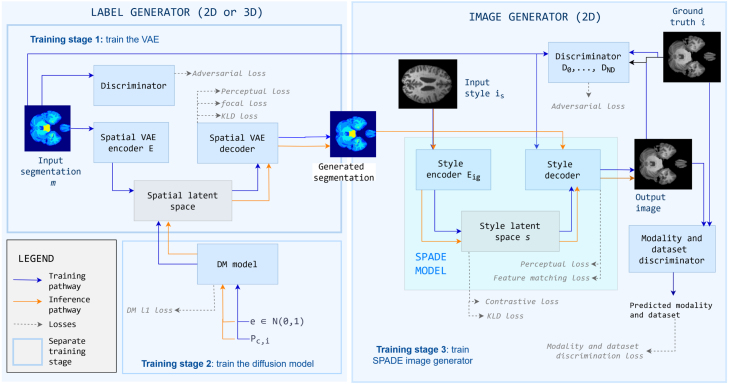
Architecture of brainSPADE. Different training stages are enclosed in blue First, we train the label generator: in the first stage, we train the VAE to reconstruct the semantic maps; then, we train the diffusion model to generate latent representations of the VAE, conditioned by the pathology proportion $P_{c,i}$. Last, the SPADE image generator is trained on unpaired real labels and multi-modal images. At inference, the user specifies $P_{c,i}$ to produce a label map (2D or 3D, depending on the approach). This map is passed to the image generator (slice by slice in the case of 3D maps) along with a 2D style image, to produce a resulting 2D image (or a stack of 2D images that can be assembled together by concatenation). Blue arrows show the training pathway and orange ones show the inference pathway. Grey dotted arrows point to the training losses.

Our model, brainSPADE, generates brain semantic label maps, including healthy and pathological regions, and corresponding multi-modal MR images. Its 2D version, brainSPADE2D was first introduced in Fernandez et al. (2022), and further modified in this work by adding conditioning on the label generator and modifying the losses in which both label and image generators were optimised. It was originally implemented as a fully 2D model comprising a label and an image generator. In this work, we extended the label generator to 3D, coupling it to a 2D image generator and resulting in 3D labels and images. We name this version “brainSPADE3D”. A diagram illustrates how brainSPADE works in Fig. 1. The architecture consists of a two-stage approach: first, a semantic label map generator based on a latent diffusion model (LDM), made up of a spatial variational autoencoder and a diffusion model operating in its latent space; second, a semantic VAE-GAN producing a realistic-looking brain image corresponding to the input semantic map and following the input contrast.

#### 4.2.1 Label generator

Unlike natural images, segmentations lack textural information, making them challenging for standard generative models such as GANs, as they are prone to instability (Goodfellow et al., 2014). We used state-of-the-art latent diffusion models (LDM) to overcome these limitations. Diffusion models (DMs) sample from a Gaussian noise distribution and denoise the sample via a Markov Chain process. Recent work has shown the generative potential of these models, as well as their training stability (Rombach et al., 2021). However, their major drawback is that they operate in the image space, making the training process slow and computationally greedy. LDMs are diffusion models that operate in a latent space: they are coupled to a spatial variational auto-encoder (S-VAE) that downsamples inputs into a compressed space. LDMs translate Gaussian noise samples into meaningful latent space samples that the auto-encoder can decode.

Our label generator is entirely based on the one from Fernandez et al. (2022), which is, in turn, based on Rombach et al. (2021). The code for the 2D label generator model was built from that of our previous work, while the one from the 3D label generator was built using MONAI Generative Models (Pinaya et al., 2023). Both follow the architecture depicted in appendix Fig. A.15, Fig. A.16.

**S-VAE**: For brainSPADE2D, we trained a spatial VAE (S-VAE) with two downsamplings and a latent space of dimension (48, 64, 3). For brainSPADE3D, we trained a spatial VAE with three downsamplings and a latent space of dimension (20, 22, 14, 8). Note that more downsamplings were required in the case of brainSPADE3D because of computational constraints. Both 2D and 3D VAEs were optimised with the following loss:

(1) $$L_{VAE}=\lambda_{KLD}L_{KLD}\left(E_{lg}\left(l\right)∥N\left(0,1\right)\right)+$$

$$\lambda_{perc}L_{perc}\left(l,\overset{ˆ}{l}\right)+\lambda_{adv}L_{adv}\left(D\left(l\right),D\left(\overset{ˆ}{l}\right)\right)+$$

$$\lambda_{fl}L_{fl}\left(l,\overset{ˆ}{l}\right)+\lambda_{nr}L_{nr}\left(l,\overset{ˆ}{l}\right)$$

where l is the input label map, $\overset{ˆ}{l}$ is the reconstructed segmentation, $L_{KLD}$ is the Kullback–Leibler divergence, $E_{lg}$ is the encoder of the VAE, $L_{perc}$ a perceptual loss, and $L_{adv}$ a patch-GAN adversarial loss, obtained by forwarding synthetic and real segmentations through a Patch-GAN discriminator D; $L_{fl}$ is a focal reconstruction loss (γ = 0.3) (Lin et al., 2018); $L_{nr}$ is a loss that minimises the focal loss between the ground truth label and the label decoded from a latent vector to which random Gaussian noise has been added (we selected μ=0;σ=0.05, as we found that, empirically, these values led to realistic noise levels). The inclusion of perceptual and adversarial losses in the training has shown a significant quality improvement in the reconstructions (Rombach et al., 2021). The network backbones used for the perceptual loss were set to VGG19 for the 2D model and MED3D (Chen et al., 2019) for the 3D model. Loss weights λ were tuned empirically and are reported in the supplementary materials (Appendix B.2).

**DM**: The 2D and 3D DM models were based on the same time-conditioned U-Net from Rombach et al. (2021). Following the objective function from Ho et al. (2020), we used a fixed noise variance between each of the 1000 timesteps and a reparametrised approach that predicts the noise added between time steps. Given a timestep t, the loss used is:

(2) $$L_{dm}=L_{2}\left(D\left(\sqrt{\bar{\alpha_{t}}}E\left(l\right)+\sqrt{1-\bar{\alpha_{t}}}\epsilon \right),\epsilon \right)$$

where $L_{2}$ is an L2 loss, D corresponds to applying the diffusion model, ϵ is a sample from a normal distribution N(0,1) of mean 0 and variance 1, $\bar{\alpha_{t}}=\prod_{i=1}^{t}\alpha_{i}$ is the cumulative variance product until time step t, with $\alpha_{t}=1-\beta_{t}$, $\beta_{t}$ being the variance at time step t, and E(l) is the latent representation of label l.

In both the 2D and 3D models, we used cross-attention conditioning on the disease proportion (WMH, non-enhancing (NE) tumour, oedema and gadolinium-enhancing (GE) tumour). For each lesion type c and image i, the lesion proportion $P_{c,i}$ was obtained by computing the number of pixels/voxels labelled as disease in the map, normalised by the maximum across the dataset, i.e.

(3) $$P_{c,i}=\frac{M_{c,i}}{max_{j}\left(M_{c,j}\right)},$$

where $M_{c,i}$ is the sum of voxels in image i corresponding to lesion c and $max_{j}$ is the maximum of this number across the dataset. In the case of the 2D model, we added an additional conditioning dimension to account for the axial slice position of our 2D semantic maps. The “relative slice number” is obtained by dividing the axial slice index (obtained during the slicing process, see Appendix B.1 for further details) by the maximum dimension across it (256 in the case of our MNI-registered images).

**Training procedure:** The 2D and 3D LDM models were trained on a set of 12 280 slices and 712 volumes, respectively, from SABRE and BraTS-TCIA (see Table 1). To strengthen the ability of our model to produce multi-pathological data, we selected a random set of 50 BraTS subjects and superimposed their tumour masks on the label maps of 50 randomly selected SABRE subjects, resulting in 50 new label maps containing both lesions. These labels were added to the 3D LDM training set. Even though BraTS and SABRE were registered to the same space, this did not always result in visually plausible images for all cases, but we hypothesised that these few samples would still help the model yield more consistent conditioning. To verify if the model could still extrapolate and produce unseen disease combinations without these extra labels, we trained an LDM with and without these 50 volumes.

#### 4.2.2 Image generator

**Original framework**: The image generator is based on SPADE (Park et al., 2019). SPADE is a semantic synthesis VAE-GAN where the style and content pathways are separate. The encoder branch $E_{ig}$ takes in an input style image i and encodes it into a compressed latent space representation s, while the decoder combines this latent style s and an input semantic map m driving the content of the output $\overset{\sim }{i}$. m is fed to the decoder via special normalisation blocks called SPADE blocks. Park et al. (2019) coupled it to a multi-scale patch-based discriminator $D=\left\{D_{0},\ldots ,D_{N_{D}}\right\}$, where $N_{D}$ is the number of individual Patch-GAN discriminators, each focusing on a different scale of the input. The original loss used to train SPADE is an addition of (1) an adversarial (GAN) loss, $L_{gan}=\sum_{j=1}^{N_{D}}HL\left(D_{j}\left(i\right),D_{j}\left(\overset{\sim }{i}\right)\right)$, where HL is the Hinge loss; (2) the VAE Kullback–Leibler divergence (KLD) loss $L_{KLD}=KLD\left(s∥N\left(0,1\right)\right)$; (3) a perceptual loss $L_{perc}=\sum_{j=1}^{M}L_{1}\left(VGG_{j}\left(i\right),VGG_{j}\left(\overset{\sim }{i}\right)\right)$, where $VGG_{j}$ are M internal representations of a VGG19 network (Johnson et al., 2016) for the ground truth image i and the synthetic one $\overset{\sim }{i}$; (4) and a feature loss $L_{feat}=\sum_{j=1}^{N_{D}}\sum_{k=1}^{k=F}L_{1}\left(D_{j,k}\left(i\right),D_{j,k}\left(\overset{\sim }{i}\right)\right)$ based on the $L_{1}$ loss between F intermediate feature representations of the multi-scale discriminator for the real and synthetic images. $L_{feat}$ aims to regularise the multi-scale discriminator.

**Modified training framework**: The original SPADE network was designed for computer vision applications, and hereby, we identified some limitations when it came to applying it to medical imaging that we tried to mitigate accordingly in our previous work Fernandez et al. (2022).

1. **Implicit latent space clustering**: Whereas the styles in computer vision are merely a qualitative concept (i.e., in a landscape dataset, a style can be the *dawn* or *night*), the contrasts in MRI are directly related to the physical tissue properties and scanner settings. Thus, we had to ensure that the same contrast from different slices of the same subject results in the same style code and that codes belonging to different acquisitions with the same modality are clustered together. Because the acquisition parameters, even for the same modality, can differ across datasets, resulting in slightly different contrasts, we also aimed to cluster the codes belonging to the same dataset together, within the modality cluster (e.g. within the ‘T1’ cluster, have a ‘T1-SABRE’ cluster a ‘T1-ADNI’ cluster etc.). For this, we implemented two losses. The first, a modality and dataset discrimination loss $L_{mod-disc}=\lambda_{mod}BCE\left(m,\overset{ˆ}{m}\right)+\lambda_{dat}BCE\left(d,\overset{ˆ}{d}\right)$, where m and d are the ground truth modality and dataset, $\overset{ˆ}{m}$ and $\overset{ˆ}{d}$ are the modality and datasets predicted on the generated images by a feed-forward network pretrained on the training dataset, and BCE is the binary cross-entropy loss. More information about this network characteristics and training are included in the implementation details in Appendix B.4. Secondly, we used a contrastive loss (Chen et al., 2020) $L_{cont}=cosim\left(E_{ig}\left(i\right),E_{ig}\left(\overset{\sim }{i}\right)\right)$, where $E_{ig}$ is the encoder of the image generator, i the input style image, $\overset{\sim }{i}$ a randomly affine-augmented version of the input style image, and cosim the cosine similarity.
2. **Binary segmentations**: the original network took in binary categorical segmentations. In Rusak et al. (2020), it was shown that using probabilistic segmentations, where a pixel is attributed probabilities of belonging to more than one tissue, yields finer results for brain image synthesis. Therefore, we replaced the categorical labels with probabilistic ones, which are also output by GIF (Cardoso et al., 2012).
3. **Paired styles and segmentations**: Originally, during training, the input style image i matched the input segmentation m fed to the decoder, making it hard to rule out whether the style image has some influence on the decoder or the segmentation on the style encoder. During training, we enforced the separation of the style and content pathways by using unpaired style images and segmentation slices within the same volume, to force disentanglement and increase the consistency across slices by forcing any input style slice to convey only the contrast. Hence, we input image $i_{s}$ to the encoder, which does not match map m, unlike ground truth image i, which also shares its contrast with $i_{s}$. We do this by drawing a different 2D image slice $i_{s}$ from the same 3D volume from which we draw the ground truth image i, which is paired to its input map m.

The two new losses $L_{mod-disc}$ and $L_{cont}$ were added during training to the original SPADE losses. The weights used for these losses and the new ones are provided in the appendix (Appendix B.3).

To balance the training and avoid mode-collapse, we implemented an adversarial scheduler, which establishes whether the discriminator and generator are trained as a function of the discriminator accuracy averaged across the last 20 iterations. Over an upper threshold $T_{up}=0.75$, only the generator is trained. On the other hand, only the discriminator is trained if its accuracy is below a lower threshold $T_{down}=0.6$. Between the two, both networks are trained. These thresholds were chosen empirically. The discriminator accuracy was calculated as an average accuracy across discriminated patches and individual discriminators. Besides concatenating the semantic label to the ground truth/synthesised image before inputting it to the discriminator, we concatenate the image modality to give the discriminator more context about the variability existing within the inputs.

### 4.3 Dataset sampling

An overview of the differences between sampling from brainSPADE2D or brainSPADE3D can be seen in the appendix.

**brainSPADE2D:** In brainSPADE2D, the user can condition the output on (1) which pathologies are present as a proportion within the slice. In cases where the user does not want a lesion to be present, the conditioning value associated with that lesion is set to 0. Otherwise, we draw it from a uniform distribution bounded by the 1st and 3rd quartiles of the disease proportion across our training dataset; (2) the slice number, as a float value <1; (3) the desired contrast, by passing an axial image with the desired MRI modality to the encoder of the image generator.

**brainSPADE3D:** Similar to brainSPADE2D, the user can condition the output on the pathology proportion in a similar manner by specifying a relative proportion of the disease within the volume. After generating a 3D label, each of its axial slices is forwarded to the image generator, one by one. Resulting axial 2D images are assembled into an output 3D volume. To ensure a consistent intensity transition between neighbouring slices, we keep the style image constant across the process.

The sampling from the label generator was accelerated using a denoising diffusion implicit model (DDIM) sampler (Song et al., 2020); the number of intermediate time steps was set to 200 across all experiments unless stated otherwise.

### 4.4 Evaluation methodology

#### 4.4.1 Label evaluation

There is no established standard for assessing label quality, as there is for images (SSIM, $FID_{Rad}$, etc.). As a surrogate of a label quality evaluation method, we compared the proportion of regions of interest within a volume or slice between our synthetic label maps and real datasets. For this purpose, we used 150 synthetic and real volumes (equivalent to ∽15 000 slices in 2D). Additionally, we studied the effect of conditioning on the output labels by observing and quantifying the different lesions when we vary the conditioning values.

#### 4.4.2 Qualitative image evaluation

In addition to providing visual examples in 2D and 3D (reconstructed volumes by forwarding volumetric segmentations slice by slice), we generated a set of 250 slices spanning multiple modalities and diseases and shuffled them into a dataset of 250 real slices, then asked a human rater with >15 years of experience in neuroimaging to label them as real or fake.

#### 4.4.3 Quantitative image evaluation

As a reference, we simulated images from the real and the synthetic labels using a similar approach to synthesis method proposed in Billot et al. (2023). For each modality m and label map L, we obtain a ‘statistics-derived’ (SD) image $SDI_{m,L}$:

(4) $$SDI_{m,L}=BF\overset{R}{\underset{i=1}{\sum }}L_{i}\times N\left(\mu_{m,d,i},\sigma_{m,d,i}/K\right)$$

where BF is a random bias field operator implemented using MONAI (range: 0.05, 0.15, probability: 0.4), N is a Gaussian distribution of mean $\mu_{m,d,i}$ and standard deviation $\sigma_{m,d,i}/K$, K is a modality-dependent factor selected empirically to ensure good contrast between tissues, and R is the number of semantic regions. The modality means and standard deviations were obtained by computing the average intensity for semantic region i and dataset d on a bias field-corrected subset of our dataset (50 images per modality and dataset). The dataset selected for each simulated image was randomly selected. Example images are displayed in Fig. 6, Fig. C.17, Fig. C.18.

We simulate T1, FLAIR and T2 SD images from the test set of 1176 label slices. We also forward these labels through our image generator, using style images from the same test dataset. For the resulting SD and generated images, we calculate the Structural Similarity Index (SSIM) and the mean square error (MSE) with regard to their ground truth. To further assess the performance of the image generator, we calculate the Frechet Inception Distance (FID) (Heusel et al., 2017) on the activations of the last functional layer of an InceptionV3 network trained on RadImageNet (Mei et al., 2022) between real training images and (we use two sets of real images $gt_{1}$ and $gt_{2}$) (1) synthetic 2D images generated from real labels ($gen_{real-labs}$) (2) SD images generated from real labels ($SD_{real-labs}$) (3) synthetic 2D images generated from synthetic 2D labels ($gen_{syn-labs-2D}$) (4) SD images generated from synthetic 2D labels ($SD_{syn-labs-2D}$) (5) synthetic 2D images generated from sliced synthetic 3D labels ($gen_{syn-labs_{3}D}$) (6) SD images generated from sliced synthetic 3D labels ($SD_{syn-labs_{3}D}$). We call this RadImageNet FID metric “$FID_{Rad}$”. As more samples are required for $FID_{Rad}$ calculation, we use 6000 T1, FLAIR and T2 images for each of these groups. For reference, we also calculate the $FID_{Rad}$ between different sets of 6000 real training images.

#### 4.4.4 Data privacy

The premise of our work focuses on creating synthetic models and datasets that can be shared. This is only the case if, indeed, the resulting images cannot be traced back to the original subjects. A validation phase should confirm this fact, but there are a number of impracticalities surrounding such a test:

• There is no specific agreement on what is considered acceptable regarding privacy in synthetic datasets. Some regulatory bodies and institutions, such as the NHS Transformation Directorate in the United Kingdom, provide broad guidelines on what synthetic data should comply with, but the research community is still far from specific metrics or methods. The literature on privacy and Generative Modelling is mainly theoretical and rarely provides practical examples (Sun et al., 2021).
• There is a diverse scenario in terms of potential data breaches. Model inversion attacks consist of retrieving the training data from the model weights. Membership attacks assume that the attacker has access to the training samples: a data breach happens when it is confirmed that those samples were indeed used to train the model (Sun et al., 2021). The few examples found in the literature for these focus on niche scenarios and thus are hardly applicable to our case.

There is, however, a consensus on the need to train these models on sufficient data to avoid overfitting (Carlini et al., 2023). We tried to avoid this by using substantial data augmentation in both the image and label generators. The full separation between the content and style streams imposed by our two-stage scenario further diminishes the probability of a sample being mapped back to a specific label-image pair. Despite the measures, we still wanted to assess whether it would be theoretically possible to know – at least – if the model is memorising the data. To do this, we focused on the following questions:

• Are the labels being memorised? We sample a certain number of labels and, for each of them, we retrieve the nearest neighbours in terms of dice score (DS) in the training dataset and plot them.
• Is the image generator behaving differently when presented with an input style from the training distribution than it does when presented with one from an unseen distribution? For this, we use images and labels coming from three 500 slice sets: (1) in-distribution training data; (2) in-of-distribution test data; (3) out-of-distribution data (from OASIS); (4) out-of-distribution data (from BraTS-OTHER). We assess whether style codes belonging to these four categories can be clustered using a K-Means algorithm (initialised at random, 300 iterations). To keep the test fair, we ruled out tumour images from the ID sets, as the OD set does not have this phenotype.

#### 4.4.5 Evaluation on segmentation tasks

The critical validation experiments must prove that our synthetic data can be employed to train segmentation models. For this, we propose several segmentation tasks in which we compare the performance of segmentation models trained, in part or exclusively, on different amounts of synthetic data to that of models trained on real data. To ensure reproducibility, we use 2D and 3D nnU-Net (Isensee et al., 2021), using the default settings, and training for 1000 epochs. All the models were tested on specific sets referred to in each experiment section. Two-sided Wilcoxon signed-rank tests where ran on the performance metrics using *scipy*, setting α to 0.05.

## 5 Results

### 5.1 Quality of the generated labels

**Fig. 2:**
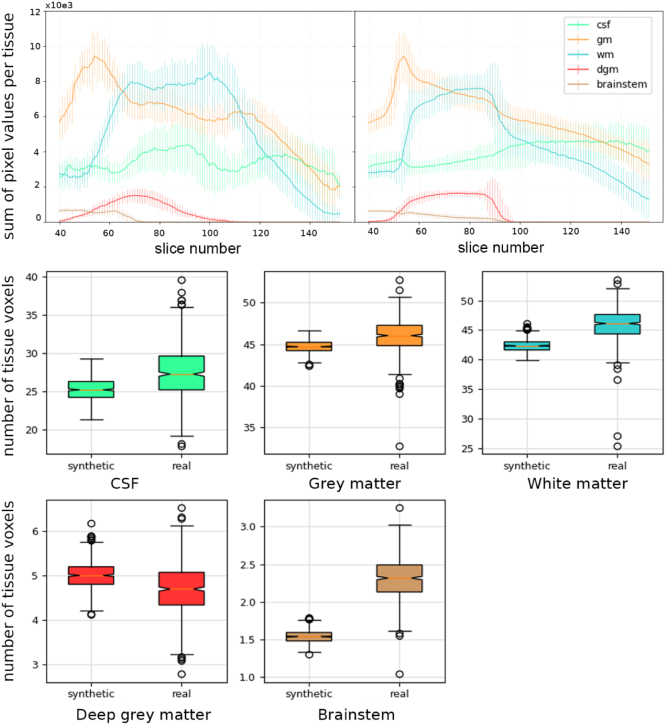
Top: sum of pixel probabilities per tissue and slice for real (left) and synthetic (right) 2D label datasets, vertical bars indicating 2× the standard deviation for each slice; bottom: sum of voxels for synthetic (left) and real (right) per tissue ($\times 10^{3}$) .

**Tissue proportions**: To evaluate the quality of the generated 2D labels, we calculated the proportion of brain pixels of each semantic region as a function of the slice number for a set of 5000 generated healthy labels and compared it to the proportions from the training set. For the 3D label generator, we calculated the total proportion of brain pixels of each semantic region. Results are shown in Fig. 2. The 2D label generator manages to approximately follow the distribution per tissue and slice, of real data, showing that the slice conditioning works effectively. In the case of the 3D label generator, we observe that the standard deviation of all synthetic tissues is statistically lower than that of real ones, showing less variability overall. In addition, we can see a slight discrepancy in the means, with the synthetic brainstem mean being considerably lower than its real counterpart.

**Fig. 3:**
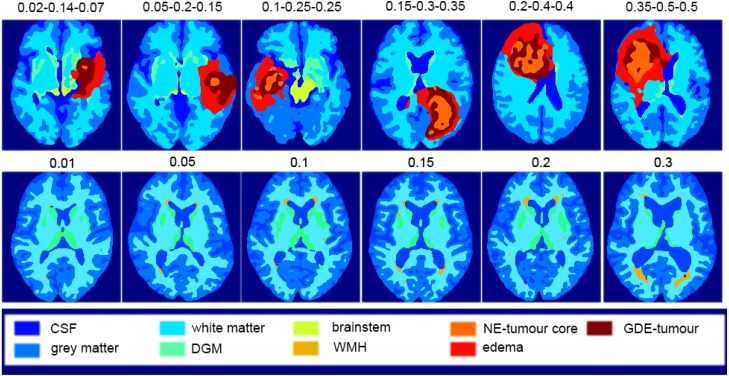
Label maps sampled from the 3D label generator using increasing conditioning for the three tumour layers (top row: necrotic and non-enhancing tumour core conditioning value, peritumoral oedema conditioning value, and GD-enhanced tumour conditioning value, separated by hyphens) and WMH (bottom row). The axial slice chosen for tumours displays the maximum tumour size. For WMH, the same middle axial slice is shown for all samples.

**Fig. 4:**
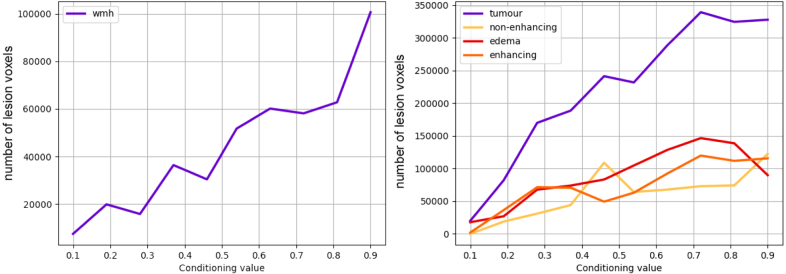
Number of lesion voxels as a function of increasing conditioning values for WMH (left) and tumour (right).

**Visual quality:** A visual assessment of real labels and images (for an example, see Fig. D.19) shows that the middle brain, where the probabilistic labels are noisier, results in many brainstem voxel probabilities to fall under 0.5 in favour of other tissues, such as white or deep grey matter, which explains the observed result. In addition, this visual assessment shows that, even though causing a minor impact on the metrics showcased by Fig. 2, there is some loss of sharpness in the 3D labels, especially in some cortical folds (which causes the outer CSF to disappear, explaining why its mean is also lower in Fig. 2) and the cerebellar cortex. The lack of sharpness can be explained by the blurring caused by autoencoders, which has been observed in the literature (Zhao et al., 2017). This is a limitation of using diffusion versus using latent space diffusion, the latter of which is essential given the memory constraints of 3D modelling. The lack of variability can be explained by limited capacity, or by the absence of any conditioning that enforces variability across healthy subjects, such as age, something that could be left for future enhancements and has already been applied to generative modelling for brain MR (Pinaya et al., 2022).

**Impact of conditioning**: To evaluate the impact of the lesion conditioning, we evaluate the size of output WMH and tumour (all three subclasses) lesions when we increase the value of their corresponding conditioning. To sample the labels we used DDIM (Song et al., 2020), with 200 steps. Fig. 3 shows that, visually, for both tumours and WMH, increasing the conditioning value results in bigger lesion sizes. This is supported by Fig. 4, where the number of voxels corresponding to a lesion tends to increase as we increase the conditioning value.

Note that we limited this experiment to the 3D model because, in the 2D label generator, the lesion proportion can be dependent on the slice number as well, which makes it hard to disentangle the lesion size and the specific slice that is generated and hard to observe the progression we can see in Fig. 3. More information about 2D lesion conditioning analysis can be found in the appendix (see Appendix D and Fig. D.20).

**Fig. 5:**
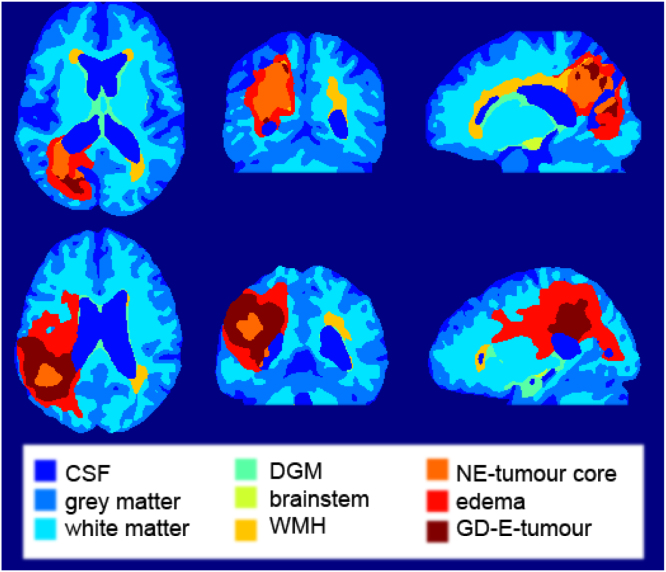
Sample label maps generated using unseen conditioning combinations (WMH + tumour), showing how the model is able to generate both lesions, with WMH showing up in yellow, and the tumour layers in dark orange-red.

**Lesion extrapolation**: We evaluated the ability of our 3D label generator to produce unseen conditioning combinations (WMH + all tumour layers). We used the model that had not seen any combination of these pathologies and used random values for all disease types. Approximately 41% of the sampled labels exhibited all of the lesions. Examples that show how the model is able to extrapolate can be seen in Fig. 5. Due to the same reasons exposed in the previous section, we limited this test to the 3D model.

### 5.2 Quality of the generated images

**Fig. 6:**
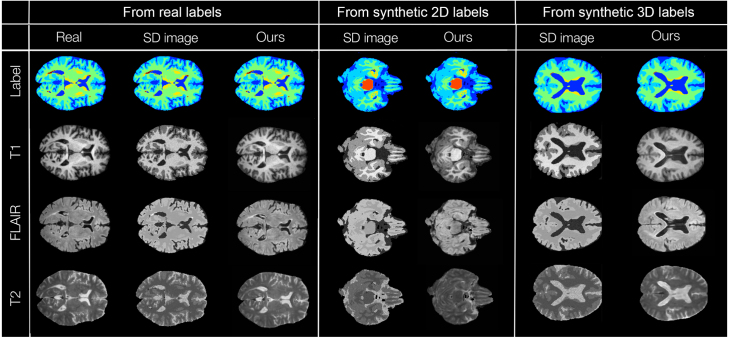
Example generated and SD (statistics-derived) T1, FLAIR and T2 images generated using real labels (left section), synthetic 2D labels (middle section) and synthetic 3D labels (right section). Ground truth images are also depicted for the images derived from real labels.

**Quantitative evaluation:** The SSIM, MSE and $FID_{Rad}$ metrics are reported in Table 2, with example images displayed in Fig. 6. For all cases (images derived from real, synthetic 2D and synthetic 3D labels) and all modalities, the images generated by our model yield a considerably higher SSIM, and lower MSE and $FID_{Rad}$ than the SD ones. Nonetheless, the $FID_{Rad}$ score obtained between different sets of real images $gt_{1}-gt_{2}$ is generally at least an order of magnitude less than the rest of the calculated $FID_{Rad}$ scores. To verify the impact of perturbations on $FID_{Rad}$, we smoothed one of our real images set ($gt_{2}$) by applying Gaussian smoothing (σ=1.0) using an operator B implemented with MONAI. Even though the blur was subtle enough that the perturbations were not noticeable with the naked eye, the $FID_{Rad}$ score ($gt_{1}-B\left(gt_{2}\right)$) was considerably affected, indicating that the slightest blur can change the $FID_{Rad}$ to values that are comparable to ($gt_{1}-gen_{syn-labs-2D},gt_{1}-gen_{syn-labs-3D})$, and even over ($gt_{1}-gen_{real-labs})$ the ones observed for our generated images. Generally, synthetic labels deteriorate the $FID_{Rad}$, especially for FLAIR and T2 images.

**Qualitative evaluation:**Table 3 shows that the scores obtained by a human rater when attempting to discern real and synthetic images are at a chance level, which indicates that the 2D images are visually indistinguishable from real ones. We did not attempt the same test with 3D images since, due to the slight inter-slice interference persisting in the reconstructed volumes, it is obvious that the images are synthetic.

**Table 2:** SSIM and MSE quality metrics between the generated - gen_real-labs_ - and statistics-derived - SD_real-labs_- T1, FLAIR and T2 images using the real labels with regards to the ground truth test set; $FID_{Rad}$ obtained between non-overlapping ground truth sets gt_1_ and gt_2_, between gt_1_ and a gt_2_ subject to a smoothing operator B, between gt_1_ and images generated from real labels gen_real-labs_ and synthetic ones gen_syn-labs_, and between gt_1_ and images derived from real labels SD_real-labs_ and synthetic ones SD_syn-labs_.

Metric	Set	T1	FLAIR	T2
SSIM	$gen_{real-labs}$	0.985_0.012_	0.978_0.017_	0.982_0.015_
	$SD_{real-labs}$	0.849_0.071_	0.855_0.061_	0.884_0.056_

MSE($\times 10^{2}$)	$gen_{real-labs}$	0.337_0.096_	0.551_0.013_	0.604_0.012_
	$SD_{real-labs}$	0.939_0.337_	0.844_0.221_	0.810_0.121_

$FID_{Rad}$				

Set				

$gt_{1}$-$gt_{2}$		0.025	0.067	0.147
$gt_{1}$-$B\left(gt_{2}\right)$		0.278	0.199	0.363
$gt_{1}$-$gen_{real-labs}$		0.100	0.037	0.340
$gt_{1}$-$SD_{real-labs}$		0.294	0.178	0.275
$gt_{1}$-$gen_{syn-labs-2D}$		0.215	0.141	0.239
$gt_{1}$-$SD_{syn-labs-2D}$		0.393	0.262	0.481
$gt_{1}$-$gen_{syn-labs-3D}$		0.137	0.099	0.124
$gt_{1}$-$SD_{syn-labs-3D}$		0.353	0.212	0.433

**Table 3:** Accuracy, precision and recall per-modality obtained by an expert on classifying real or fake slices.

Contrast	Accuracy	Precision	Recall
T1	0.490	0.489	0.440
FLAIR	0.540	0.530	0.700
T2	0.420	0.433	0.520

### 5.3 Ablation study

**Fig. 7:**
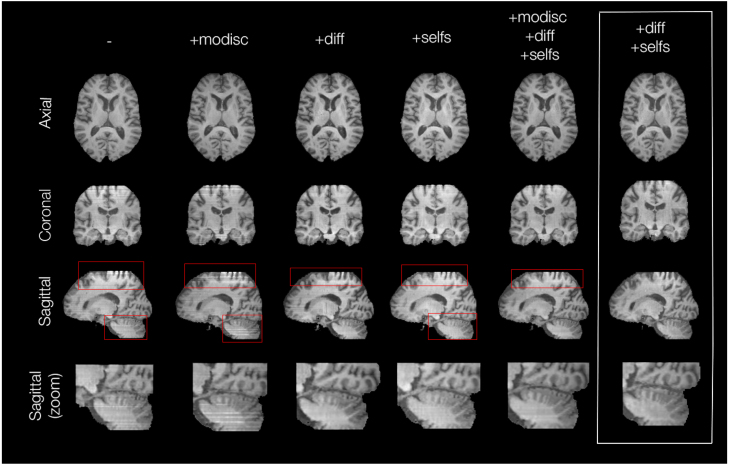
Example reconstructed T1 volumes. From top to bottom: axial, coronal, sagittal slices and zoom on the cerebellum of the sagittal slices of the volumes reconstructed from the stack of synthetic T1 slices. From left to right: models using no additional losses (original SPADE network), $L_{mod-disc}$ loss only (+modisc), unpaired style and semantic slices (+diff), $L_{cont}$ loss only (+selfs), all losses and unpaired style and semantic slices (+modisc, +diff, +selfs), and the statistically best one, using $L_{cont}$ and unpaired style and semantics slices (+diff, +selfs). Red frames show areas of higher inconsistencies between 2D slices.

To assess the impact of the additional losses or features covered in Section 4.2.2, we ran an ablation study (for more details refer to Appendix C). We evaluated the consistency between generated neighbouring slices. Whereas the modality discrimination loss did not seem to qualitatively or quantitatively ameliorate consistency, the use of unpaired styles and segmentations and the contrastive loss significantly improved the inter-slice structural similarity index and the visual consistency, for T1 and FLAIR images especially. This can be observed in Fig. 7 (FLAIR and T2 examples are available in the appendix - Fig. C.17, Fig. C.18). Hence, we removed the modality and dataset discrimination losses $L_{mod-disc}$ introduced in Section 4.2.2-**Modified training framework** in further trainings.

### 5.4 Code clustering

**Fig. 8:**
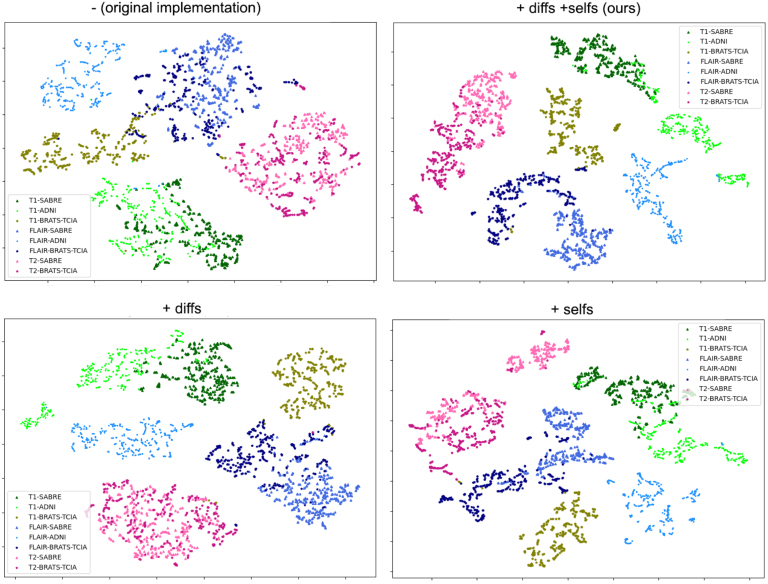
t-SNE plots obtained on the test set codes for the model trained with no additional losses and paired style and label slices (top left), using both contrastive loss and unpaired slices (top right, ‘+diff +selfs’), using different slices (bottom left, ‘+diff’) and contrastive loss (bottom right, ‘+selfs’). The colours denote different modalities and datasets.

To assess the impact of the selected losses and added features ($L_{cont}$ + using unpaired slices) on the style latent space, which aimed to cluster the style representations, or codes, of different 2D slices belonging to the same modality and dataset together, and remove any influence from the semantic content of these slices, we studied whether these styles were clustered as a function of their modality and dataset. For that, we first reduced the dimension of the 16-dimensional latent space codes to 2 dimensions using t-stochastic neighbour embedding (t-SNE, perplexity: 15, learning rate: 400). We encoded the images belonging to our test set introduced in Section 4.4.3, ran t-SNE and plotted the resulting 2D codes, for the models trained with contrastive loss only, with unpaired slice selection only, with both of these features, and with neither (see Fig. 8). Whereas the model trained on only the original SPADE losses displays some modality clustering, with blue (FLAIR), pink (T2) and green (T1) clusters being visible, there is more overlap between the clusters than in the others, notably between FLAIR and T1 images belonging to BRATS. In addition, our model achieves more clustering between codes belonging to the same dataset (within each modality), something that is likely translating into more style consistency.

### 5.5 Assessment of data memorisation

**Fig. 9:**
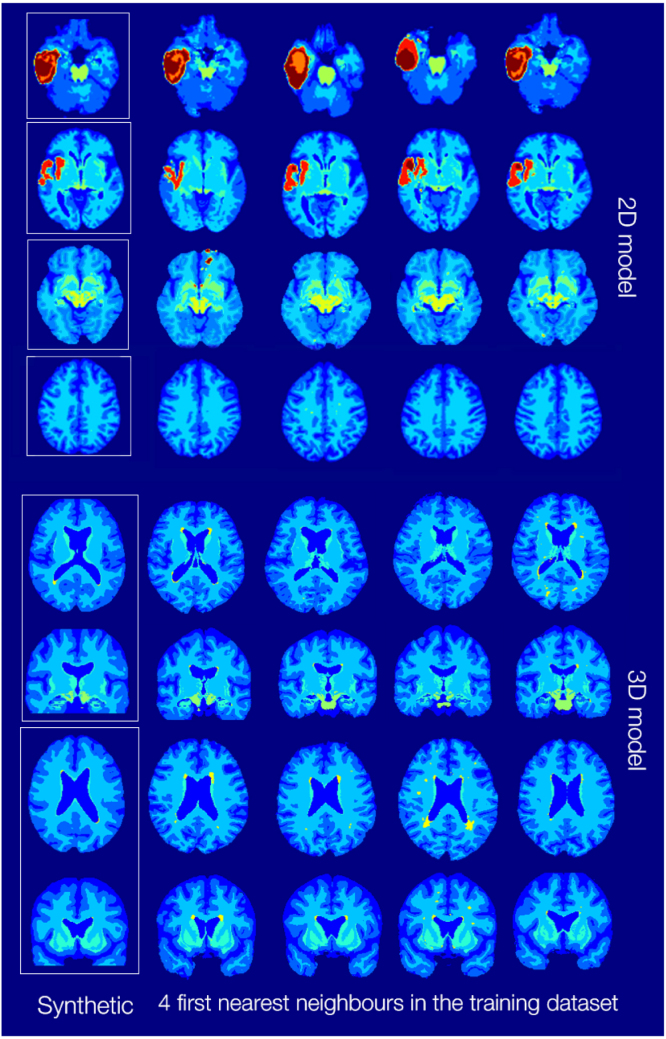
Sample 2D and 3D synthetic labels, and their four nearest neighbours in the training dataset. For the 3D images, axial and coronal views are provided.

**Memorised labels:** As discussed in Section 4.4.4, we assessed whether the label generator was memorising samples. For this, we sample a series of labels using various types of conditioning, and perform qualitative comparisons between these samples and their four nearest neighbours (NN) in the training dataset, using Dice score as a ranking metric. Examples are shown in Fig. 9. All examined samples showed differences with regard to their NNs, suggesting that the model is likely not memorising the data. The closest similarities are those between the generated tumours (for example, the second row in Fig. 9). This is in line with the observations made by Carlini et al. (2023), where diffusion models were more likely to memorise out-of-distribution images. Indeed, even though we had a substantial amount of tumour volumes, they still have a relatively small prevalence within the training dataset — especially in 2D, where only a small fraction of slices from a volume containing a tumour will actually have a tumour-. In addition, in 2D, the (slice number, lesion) pairing might be causing the subset of images falling under the specific conditioning to be even smaller.

**Fig. 10:**
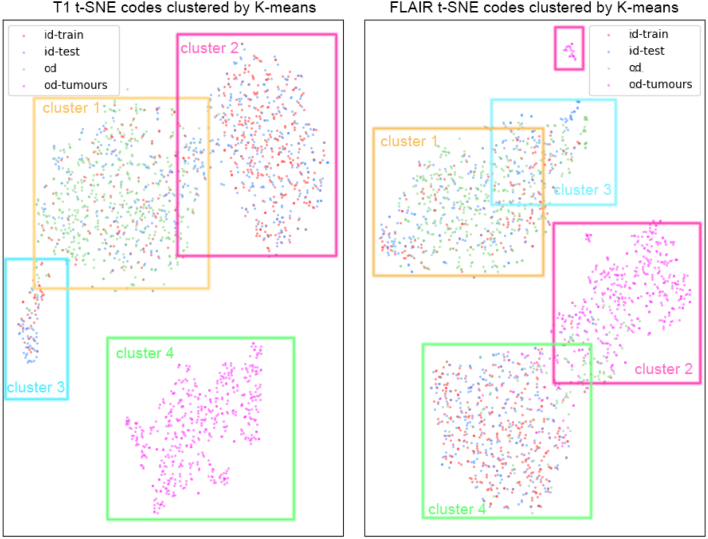
t-SNE-reduced codes for T1 (left) and FLAIR (slices) for ID-train data, ID-test data, OD data and OD data coming from BraTS-OTHER. K-means clusters are shown as coloured frames. Clusters names are assigned arbitrarily. K-Means clusters of T1 and FLAIR codes.

**Memorised image styles:** As discussed earlier, we aimed to see whether the style encoder of our image generator is overfitting to the training distribution, in a way that makes it possible to retrieve sample slices that were part of the training set. For this, we obtained the style latent representations for in-distribution training and test data (ID-train, ID-test), and OASIS and BraTS-OTHER out-distribution data (OD, OD-tumours), reduced them to 2D with t-SNE, and used K-Means to cluster them. Fig. 10 shows that the K-Means clusters (represented by coloured frames) differ from the distribution clusters, with the exception of the one corresponding to BraTS-OTHER, for both modalities. This showcases that the model is not memorising training data, as it does not exhibit different behaviour for training and unseen test ID data, and OD data. Even if the style representations associated with BraTS-OTHER (OD-tumours) are clearly separable from the rest, this still does not allow retrieving latents associated with training samples from the total set of latents.

### 5.6 Segmentation experiments

#### 5.6.1 Segmentation of healthy regions

This task focuses on segmenting five healthy tissues: CSF, GM WM, DGM and brainstem. We train the following nnU-Net models:

• $H_{ID-real-2D}$ Trained on ∽15 000 label slices from SABRE and T1 images from the SABRE dataset (resulting from slicing 150 volumes)
• $H_{ID-real-3D}$ Trained on 150 label volumes and T1 images from the SABRE dataset.
• $H_{ID-syn-2D}$ Trained on ∽15 000 synthetic label slices and T1 images generated using SABRE slices as style input.
• $H_{ID-syn-3D}$ Trained on 150 synthetic healthy label volumes and T1 images generated using SABRE slices as style input.

The term “ID” - “in-of-distribution” - encompasses synthetic data produced using (1) seen phenotypes (e.g. healthy, exclusive WMH or tumour lesions) and (2) seen styles (input style images belonging to datasets and modalities that have been seen during training). We tested the models on a test set of 30 T1 volumes from a hold-out set of SABRE. For the 2D models, we calculated the Dice metric after aggregating the individually segmented slices. Dice scores on each tissue are reported in Table 4. Although models trained on real data perform significantly better (p-value < 0.05) than their synthetic counterparts, we observe a comparable performance for all five tissues. Note that the higher performance of 2D real models over the 3D is likely due to a capacity issue, as the ratio between the number of model parameters and available samples was much higher for the training dataset.

**Table 4:** Mean dice scores obtained on 2D and 3D healthy ID segmentation experiments. An asterisk indicates statistical significance.

Region	$H_{ID-real-2D}$	$H_{ID-syn-2D}$	$H_{ID-real-3D}$	$H_{ID-syn-3D}$
CSF	0.952_0.007_*	0.930_0.013_	0.933 _0.008_*	0.906_0.013_
GM	0.956_0.004_*	0.933_0.010_	0.935_0.006_*	0.913_0.012_
WM	0.969_0.004_*	0.952_0.010_	0.961_0.004_*	0.943_0.006_
DGM	0.868_0.019_*	0.811_0.040_	0.805_0.026_*	0.746_0.026_
Brainstem	0.952_0.021_*	0.934_0.017_	0.931_0.019_*	0.884_0.011_

#### 5.6.2 Addressing out-distribution data with synthetic data

To investigate the ability of our synthetic data to address out-distribution domains, we focused on two tasks:

• near out-distribution (n-OD): testing synthetic data on 30 T1 volumes from ABIDE (see Table 1), provided we only have real T1 volumes from SABRE.
• far out-distribution (f-OD): testing synthetic data on 30 FLAIR volumes from OASIS, provided we only have T1 volumes from SABRE.

The regions to segment are the same as in Section 5.6.1. In the first case, we are exploring cross-dataset style transfer, whereas in the second, we address cross-modality style transfer. We sampled 2D (15 000 slices) and 3D (150 volumes) datasets conditioning on the styles of the target domains. We test in-distribution (ID) models $H_{ID-real-2D}$, $H_{ID-real-3D}$, $H_{ID-syn-2D}$ and $H_{ID-syn-3D}$ from Section 5.6.1 in both n-OD and f-OD sets. In addition, we trained $H_{n-OD-syn-2D}$, $H_{n-OD-syn-3D}$, $H_{f-OD-syn-2D}$ and $H_{f-OD-syn-3D}$ on the synthetic 2D and 3D n-OD and f-OD datasets. For reference, we also trained $H_{n-OD-real-2D}$, $H_{n-OD-real-3D}$, $H_{f-OD-real-2D}$ and $H_{f-OD-real-3D}$ on a small set of 10 T1 volumes/1090 slices from the n-OD and f-OD. A summary of these experiments and models is reported in Table 5.

Results are visible in Table 6. For n-OD, $H_{n-OD-syn-2D}$ and $H_{n-OD-syn-3D}$ typically perform better for all regions, only excelled by the reference models $H_{n-OD-real-2D}$ and $H_{n-OD-real-3D}$. Interestingly, whereas $H_{ID-real-2D}$ achieves comparable performance for CSF, WM and DGM, in 3D, $H_{ID-syn-3D}$ seems to perform better, overall, than $H_{ID-real-3D}$, showing that, in 3D, synthetic data, even sampled using ID styles, seem to help the model generalise better to unseen distributions. For f-OD, $H_{f-OD-syn-2D}$ and $H_{f-OD-syn-3D}$ perform significantly better than the ID ones, especially for DGM and brainstem, as their appearance is very different, less pronounced in FLAIR images compared to T1.

**Table 5:** Summary of models trained on OD experiment. S: synthetic/R: real. Note that the real datasets do not need a “style” per se, as they are obtained using the original images. The populated field “style” in this case refers to the dataset and modalities used to create these training sets.

2D model	3D model	R/S	style
Used in near-OD and far-OD experiments			

$H_{ID-real-2D}$	$H_{ID-real-3D}$	R	SABRE (T1)
$H_{ID-syn-2D}$	$H_{ID-syn-3D}$	S	SABRE (T1)

Used in near-OD experiment			

$H_{n-OD-real-2D}$	$H_{n-OD-real-3D}$	R	ABIDE (T1)
$H_{n-OD-syb-2D}$	$H_{n-OD-syn-3D}$	S	ABIDE (T1)

Used in far-OD experiment			

$H_{f-OD-real-2D}$	$H_{f-OD-real-3D}$	R	OASIS (FLAIR)
$H_{f-OD-syb-2D}$	$H_{f-OD-syn-3D}$	S	OASIS (FLAIR)

**Table 6:** Results for the 2D and 3D near and far out-distribution experiments. * indicates significantly better performance among all models, ** indicates significantly better performance among all models minus the reference ones (real-OD).

Dimension	Model	CSF	GM	WM	DGM	Brainstem
Near out-of-distribution						

2D	$H_{ID-real-2D}$	$0.806_{0.049}$**	$0.877_{0.045}$	$0.919_{0.031}$*	$0.700_{0.286}$	$0.799_{0.147}$
	$H_{ID-syn-2D}$	$0.768_{0.032}$	$0.877_{0.027}$	$0.918_{0.022}$	$0.690_{0.241}$	$0.793_{0.172}$
	$H_{n-OD-syn-2D}$	$0.800_{0.028}$	$0.892_{0.020}$**	$0.924_{0.019}$*	$0.789_{0.048}$**	$0.854_{0.051}$**
	$H_{n-OD-real-2D}$	$0.895_{0.018}$*	$0.940_{0.007}$*	$0.961_{0.006}$*	$0.889_{0.014}$*	$0.918_{0.034}$*

3D	$H_{ID-real-3D}$	$0.630_{0.079}$	$0.786_{0.043}$	$0.817_{0.043}$	$0.636_{0.182}$	$0.641_{0.308}$
	$H_{ID-syn-3D}$	$0.789_{0.038}$**	$0.861_{0.025}$**	$0.906_{0.020}$	$0.770_{0.042}$	$0.680_{0.240}$
	$H_{n-OD-syn-3D}$	$0.771_{0.034}$	$0.857_{0.018}$	$0.908_{0.017}$*	$0.780_{0.038}$**	$0.723_{0.245}$*
	$H_{n-OD-real-3D}$	$0.911_{0.019}$*	$0.947_{0.008}$*	$0.967_{0.007}$*	$0.906_{0.014}$*	$0.898_{0.049}$*

Far out-of-distribution						

2D	$H_{ID-real-2D}$	$0.663_{0.104}$	$0.459_{0.049}$	$0.486_{0.177}$	$0.055_{0.056}$	$0.014_{0.043}$
	$H_{ID-syn-2D}$	$0.659_{0.111}$	$0.497_{0.036}$	$0.373_{0.179}$	$0.092_{0.043}$	$0.036_{0.090}$
	$H_{f-OD-syn-2D}$	$0.740_{0.106}$*	$0.772_{0.064}$*	$0.804_{0.076}$*	$0.625_{0.105}$*	$0.841_{0.100}$*
	$H_{f-OD-real-2D}$	$0.783_{0.114}$**	$0.812_{0.071}$**	$0.843_{0.083}$**	$0.678_{0.137}$**	$0.867_{0.092}$**

3D	$H_{ID-real-3D}$	$0.683_{0.104}$	$0.546_{0.058}$	$0.583_{0.093}$	$0.093_{0.063}$	$0.062_{0.129}$
	$H_{ID-syn-3D}$	$0.697_{0.119}$	$0.468_{0.083}$	$0.472_{0.137}$	$0.043_{0.037}$	$0.012_{0.038}$
	$H_{f-OD-syn-3D}$	$0.732_{0.093}$*	$0.756_{0.059}$*	$0.790_{0.062}$*	$0.583_{0.094}$*	$0.824_{0.129}$*
	$H_{f-OD-real-3D}$	$0.825_{0.112}$**	$0.837_{0.078}$**	$0.870_{0.084}$**	$0.693_{0.128}$**	$0.858_{0.113}$**

#### 5.6.3 Disease segmentation with synthetic data

**White matter hyperintensities segmentation**:

We sampled 2D and 3D datasets conditioning the labels on only WMH, and using the style from SABRE FLAIR images as style conditioning, resulting in datasets of size 15 000 and 150 for 2D and 3D respectively, that we used to train models $D_{WMH-syn-2D}$ and $D_{WMH-syn-3D}$. We compared the performance on real datasets $D_{WMH-real-2D}$ and $D_{WMH-real-3D}$ of the same sizes as their synthetic counterparts. Additionally, since synthetic data will be potentially of use when there is not sufficient real data, we explored the potential of synthetic data to complement small datasets. For this, we trained segmentation models $D_{WMH-small-2D}$ and $D_{WMH-small-3D}$ on a subset of five subjects (for 2D: all slices corresponding to these five subjects) from the real dataset. Lastly, we combined the small real datasets and the synthetic ones into hybrid models $D_{WMH-hybrid-2D}$ and $D_{WMH-hybrid-3D}$. Results are reported in Table 7.

For most metrics and in 2D and 3D, the models trained on large real datasets perform significantly better (p-value < 0.05). In 2D and 3D, the hybrid model is able to boost the Dice and recall with regards to the models trained on scarce data, in most cases being significantly better than them (p-value < 0.05).

Note that the synthetic model in 3D achieves significantly lower Dice, precision and recall than the other models, showcasing that a synthetic-real domain gap persists in 3D.

**Tumour**: We sampled 2D and 3D datasets conditioning on the three tumour labels from the BraTs challenge and obtained T1, FLAIR and T2 images using the styles of our target distribution (the set of other sites from BraTs). We trained nnU-nets to segment all three tumour tissues: peritumoral oedema, necrotic and non-enhancing tumour core, and GD-enhanced tumour on our synthetic 2D and 3D datasets of sizes 15 000 and 150, respectively, resulting in models $D_{tum-syn-2D}$ and $D_{tum-syn-3D}$. We compared with real counterparts of equivalent sizes $D_{tum-real-2D}$ and $D_{tum-real-3D}$. Additionally, we explored the effect of combining a small labelled target dataset with our synthetic datasets. For this, we use a holdout set of six BraTS-OTHER subjects (in 2D, resulting in 1023 slices), and train segmentation models $D_{tum-small-2D}$ and $D_{tum-small-3D}$. We combine our synthetic datasets with the latter into datasets that we used to train models $D_{tum-hybrid-2D}$ and $D_{tum-hybrid-3D}$. All models were tested on a test BraTS-OTHER set of 26 subjects. Using the same evaluation as in Isensee et al. (2021), we merged the labels and computed the Dice score on (1) the whole tumour (oedema + necrotic and non-enhancing tumour core + GD-enhanced tumour) and (2) the tumour core (necrotic and non-enhancing tumour core + GD-enhanced tumour). We left out the calculation of the performance on the segmentation of solely the GD-tumour, as, since we did not use the GD-enhanced T2 images from the BraTs dataset, none of the available contrasts offer sufficient distinction between both tumour tissues. Results are reported in Table 8.

**Fig. 11:**
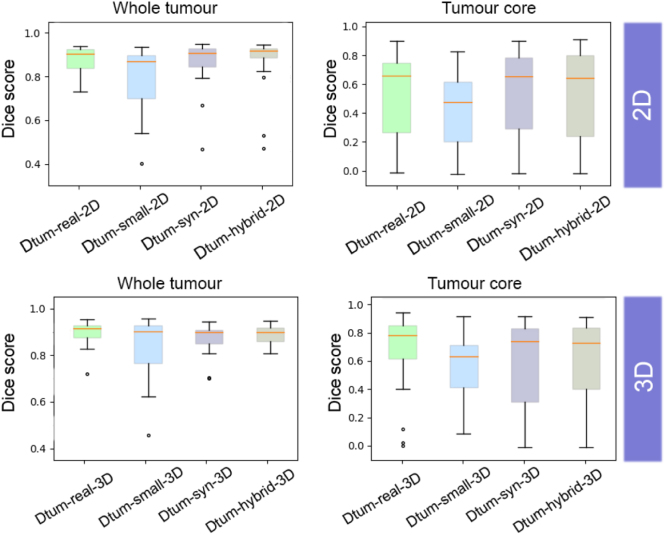
Dice distributions of the 2D (top) and 3D (bottom) tumour segmentation experiments on the whole tumour (left) and core (right).

In 2D, the model $D_{tum-small-2D}$ performed significantly worse than its counterparts on segmenting the whole tumour (p-value < 0.05). Nonetheless, no significant difference was found in the task of segmenting the tumour core due to the high variance of the performance across the test set. If we look at the Dice distributions for 2D (see Fig. 11), we can see that, for the whole tumour segmentation, the hybrid model has a lower range and higher median than its fully synthetic counterpart and $D_{tum-real-2D}$.

In 3D, we observe a similar trend as in 2D, with $D_{tum-hybrid-3D}$ being close to $D_{tum-real-3D}$ in performance for both the whole tumour and the tumour core, significantly better than $D_{tum-syn-3D}$ and $D_{tum-small-3D}$ for the whole tumour. As in 2D, the high standard deviation of the performances on the tumour core diminishes any statistical significance.

**Table 7:** Mean dice score, precision and recall obtained on 2D and 3D WMH segmentation. * indicates statistical difference in performance (if more than one model displays it, no statistical difference in performance was found between them); ** indicates statistically lower than model(s) labelled with ‘*’, but better than the rest.

Model	Dice	Precision	Recall
$D_{WMH-real-2D}$	0.787_0.070_*	0.831_0.103_*	0.761 _0.089_*
$D_{WMH-small-2D}$	0.749_0.076_	0.829_0.102_*	0.695_0.101_
$D_{WMH-hybrid-2D}$	0.759_0.073_**	0.814_0.095_	0.723_0.100_**
$D_{WMH-syn-2D}$	0.745_0.078_	0.764_0.118_	0.746_0.099_*

$D_{WMH-real-3D}$	0.806_0.070_*	0.824_0.98_**	0.801_0.095_*
$D_{WMH-small-3D}$	0.720_0.081_	0.850_0.109_*	0.637_0.110_
$D_{WMH-hybrid-3D}$	0.747_0.075_**	0.819_0.113_**	0.702_0.109_**
$D_{WMH-syn-3D}$	0.626_0.113_	0.604_0.129_	0.679_0.147_**

**Table 8:** Dice and standard deviation on Dice for the 2D and 3D tumour segmentation experiments; * indicates statistical difference in performance (if more than one model displays it, no statistical difference in performance was found between them).

Model	Dice (whole)	Dice (core)
$D_{tum-real-2D}$	0.879_0.059_*	0.559_0.275_
$D_{tum-small-2D}$	0.799_0.445_	0.455_0.243_
$D_{tum-syn-2D}$	0.872_0.100_**	0.559_0.306_
$D_{tum-hybrid-2D}$	0.876_0.114_*	0.535_0.323_

$D_{tum-real-3D}$	0.893_0.048_*	0.686_0.268_
$D_{tum-small-3D}$	0.832_0.128_	0.556_0.234_
$D_{tum-hybrid-3D}$	0.884_0.039_	0.606_0.313_
$D_{tum-syn-3D}$	0.867_0.061_	0.589_0.316_

#### 5.6.4 Impact of synthetic dataset size segmentation performance

To observe the impact of synthetic dataset size on the performance of segmentation models, we ran a separate healthy and lesion segmentation experiment varying the synthetic dataset size. For 2D models, we sampled datasets of size 50 000, 5000, 500 and 50; for 3D models, we sampled 1000, 100 and 10 pairs of segmentations and T1 images for healthy segmentation, FLAIR images for WMH segmentation and T1, FLAIR and T2 images for tumour segmentation. The segmentation models were trained, and then tested on real data, in the same fashion as in Sections 5.6.1, 5.6.3. These models were named labelled $H_{dim-S}$ for healthy segmentation, $D_{dim-S-WMH}$ and $D_{dim-S-tum}$ for WMH and tumour segmentation, with dim being the dimension, 2D or 3D, and S being the size (50K, 5K, 500, 1000, 100 or 10). Because the segmentation of healthy tissues is easier in 3D, even with small dataset sizes, we turned off data augmentation for healthy 3D models. The results obtained for healthy segmentation are reported in Table 9, and those for lesion segmentation in Table 10. Almost consistently across dimensions and tasks, the two largest datasets (1000–100 for 3D and 50 000 and 5000 for 2D) perform significantly better than the rest, except for tumour core segmentation and CSF in 3D, with the largest dataset outperforming the second largest for most tasks, notably for brainstem (2D and 3D) and DGM (3D), WMH (2D and 3D) and whole tumour (3D) showing general – but mild – improvement of segmentation scores with dataset size, especially for more complex tasks, and for 2D cases, where more samples mean more slice coverage and, likely, more relevant samples. Interestingly, significantly best performance was shared on multiple occasions by the largest and second-largest datasets, suggesting that the expressive power of the generative model might wane slightly as the synthetic dataset size exceeds that of the dataset used to train the generative model.

**Table 9:** Mean Dice scores obtained on 2D and 3D healthy region segmentation experiments with varying dataset sizes. A single asterisk indicates that the model performs significantly better (p-value < 0.05) than the rest; while double asterisks indicate that the model performs significantly better than any other model but worse than the single asterisk models. When more than one model is better, these models were not performing significantly differently between themselves.

Model	CSF	GM	WM	DGM	Brainstem
$H_{2D-50K}$	$0.932_{0.012}$*	$0.935_{0.010}$*	$0.952_{0.010}$*	$0.810_{0.038}$*	$0.934_{0.018}$*
$H_{2D-5K}$	$0.931_{0.012}$**	$0.934_{0.011}$*	$0.952_{0.010}$*	$0.810_{0.039}$*	$0.933_{0.017}$**
$H_{2D-500}$	$0.927_{0.012}$	$0.930_{0.011}$	$0.949_{0.010}$	$0.787_{0.047}$	$0.922_{0.016}$
$H_{2D-50}$	$0.925_{0.010}$	$0.927_{0.011}$	$0.945_{0.012}$	$0.754_{0.063}$	$0.896_{0.018}$

$H_{3D-1000}$	$0.869_{0.017}$	$0.885_{0.010}$**	$0.927_{0.006}$*	$0.734_{0.034}$*	$0.871_{0.014}$*
$H_{3D-100}$	$0.885_{0.015}$*	$0.891_{0.009}$*	$0.922_{0.007}$**	$0.720_{0.044}$**	$0.859_{0.015}$**
$H_{3D-10}$	$0.873_{0.019}$**	$0.883_{0.009}$	$0.915_{0.005}$	$0.711_{0.027}$	$0.850_{0.016}$

**Table 10:** Mean Dice scores obtained on 2D and 3D lesion segmentation experiments with varying dataset sizes. A single asterisk indicates that the model performs significantly better (p-value < 0.05) than the rest; while double asterisks indicate that the model performs significantly better than any other model but worse than the single asterisk models. When more than one model is better, these models were not performing significantly differently between themselves.

Tumour segmentation task		
Model	Whole tumour	Tumour core
$D_{2D-50K-tum}$	$0.857_{0.105}$*	$0.561_{0.256}$*
$D_{2D-5K-tum}$	$0.869_{0.081}$*	$0.512_{.289}$**
$D_{2D-500-tum}$	$0.849_{.089}$	$0.249_{0.194}$
$D_{2D-50-tum}$	$0.081_{.099}$	$0.074_{0.125}$

$D_{3D-1000-tum}$	$0.859_{0.069}$*	$0.577_{0.329}$
$D_{3D-100-tum}$	$0.849_{0.073}$**	$0.549_{0.278}$
$D_{3D-10-tum}$	$0.794_{0.089}$	$0.562_{0.201}$

WMH segmentation task		

Model	WMH	

$D_{2D-50K-WMH}$	$0.746_{0.074}$*	
$D_{2D-5K-WMH}$	$0.736_{0.080}$**	
$D_{2D-500-WMH}$	$0.726_{0.089}$	
$D_{2D-50-WMH}$	$0.704_{0.108}$	

$D_{3D-1000-WMH}$	$0.616_{0.116}$*	
$D_{3D-100-WMH}$	$0.593_{0.130}$**	
$D_{3D-10-WMH}$	$0.573_{0.130}$	

#### 5.6.5 Testing on synthetic data

So far, we have evaluated whether synthetic data can be used to train models and achieve acceptable performance on real data. In this small section, we test a series of models trained on real 2D and 3D data on synthetic data, to further evaluate the quality of the synthetic label and image pairs. For this, we test the following models of the previous sections on synthetic test sets:

• $H_{ID-real-2D}$ (see Section 5.6.1), we test it on 500 2D synthetic T1 images and their CSF, GM, WM, DGM and brainstem labels.
• $H_{ID-real-3D}$ (see Section 5.6.1), we test it on 50 3D synthetic T1 images and their CSF, GM, WM, DGM and brainstem labels.
• $D_{WMH-real-2D}$ (see Section 5.6.3), we test it on 500 2D synthetic FLAIR images and their WMH lesions.
• $D_{WMH-real-3D}$ (see Section 5.6.3), we test it on 50 3D synthetic FLAIR images and their WMH lesions.
• $D_{tum-real-2D}$ (see Section 5.6.3), we test it on 500 2D synthetic T1, FLAIR and T2 images and their tumour layers segmentations.
• $D_{tum-real-3D}$ (see Section 5.6.3), we test it on 50 3D synthetic T1, FLAIR and T2 images and their tumour layers segmentations.

**Fig. 12:**
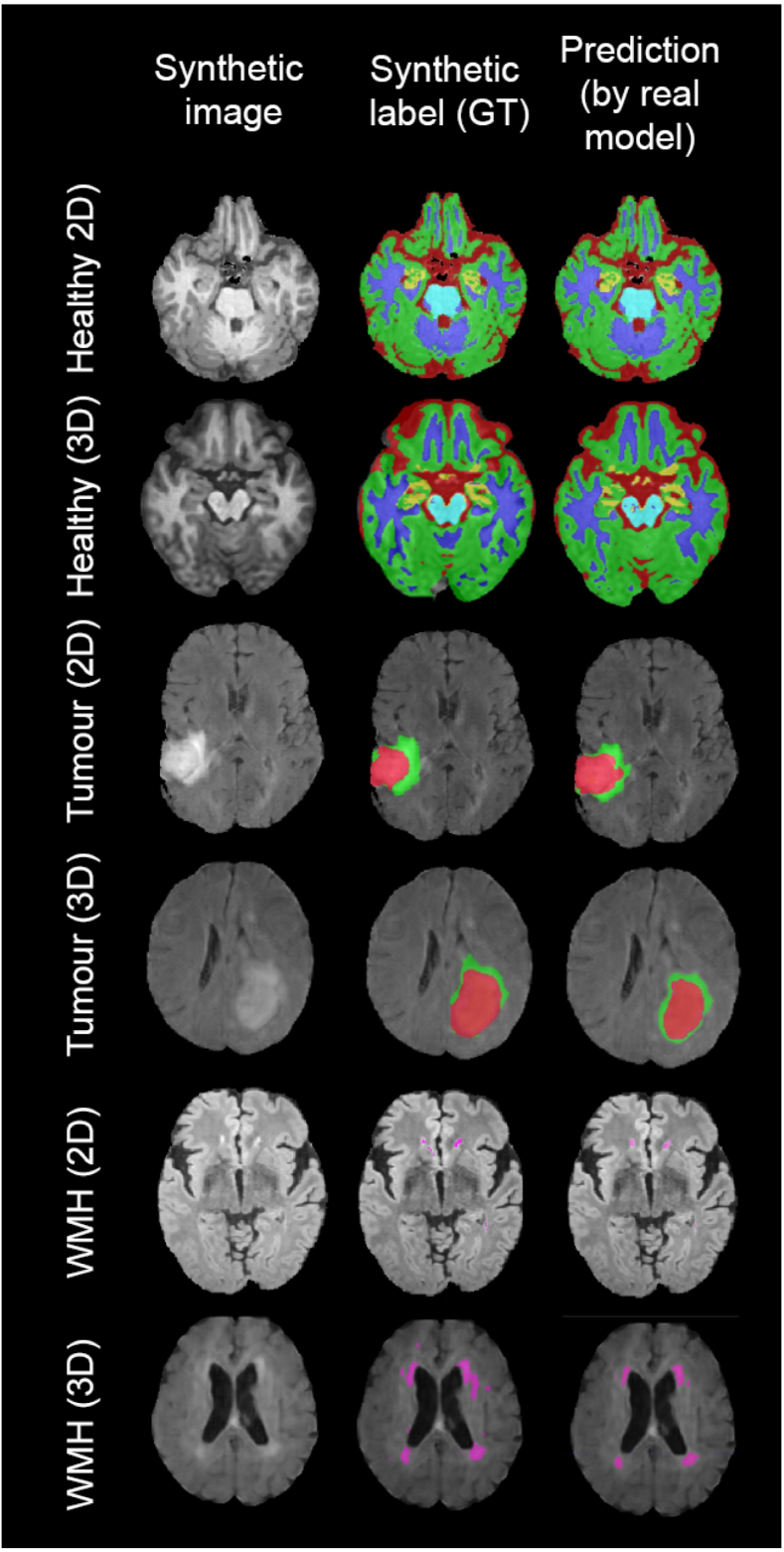
Results from experiment 5.6.5: example synthetic 2D and 3D (axial slice from 3D volume) test images, ground truth synthetic labels of healthy regions – CSF (red), GM (green), WM (blue), DGM (yellow) and brainstem (light blue) – tumour core (red) and oedema (green) and WMH (magenta), and predictions obtained by models trained on real data.

For 2D models, we cannot obtain Dice scores on aggregated 3D volumes, as we have done in previous experiments, because synthetic 2D labels are not part of a 3D volume. To have a reference for lesions and tumours, where volume Dice tends to make a difference over 2D slice Dice, we calculated the Dice obtained by the above-mentioned models on 2D slices from test sets of real data, yielding a Dice of $0.563_{0.297}$ for WMH, and $0.782_{0.321}$ and $0.451_{0.386}$ on the whole tumour and tumour core. The results of the test on synthetic data can be seen in Table 11. Visual examples of synthetic test images and segmentations are depicted in Fig. 12 Values obtained for 3D models can be compared to those reported in Tables 4, Table 8, Table 7. Both in 3D and in 2D, we do not observe a considerable difference when it comes to segmenting healthy data. For lesions, models achieve a Dice that is 15 to 25% lower on synthetic pairs than on real pairs. Despite the observed disparity between real and synthetic data pairs – especially for lesion 3D data, which can be noted visually by looking at the images from Fig. 12 –, these results show that, overall, synthetic images are consistent with their labels.

#### 5.6.6 Label extrapolation in 3D: combining pathologies into the same subject

**Fig. 13:**
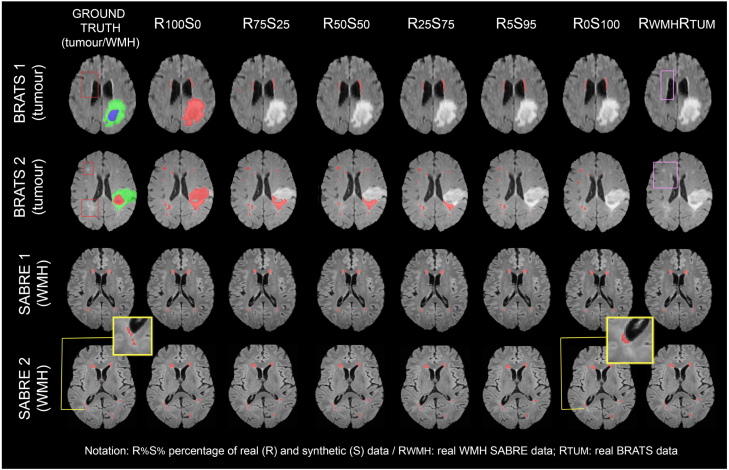
Example segmentations obtained for experiment 5.6.6 on two BraTS-OTHER subjects (top two rows) and two SABRE subjects (bottom two rows), with their respective ground truth tumour (tumour sub-layers in red, green and blue)/WMH masks (in red) displayed, displayed in the first column, and predicted WMH masks in subsequent columns (in red). Yellow frames zoom on areas where R_0_S_100_ undersegments WMH lesions. Red frames on the BraTS images show example WMH lesions (for which we do not have ground truth segmentations). Purple frames on the last column highlight the false negatives yielded by R_WMH_R_TUM_.

One of the main motivations for having a generative model of semantic labels and images is to extrapolate to unseen phenotypes. If we take the model $D_{WMH-real-3D}$ from Section 5.6.3, and now test it on the hold-out set from BraTS used in the previous tumour segmentation experiment, we obtain a high number of false positive voxels (see column two, top two rows in Fig. 13). By leveraging our model’s ability to produce paired pathological labels and images, we produce a synthetic 3D dataset containing 150 labels and images, randomising WMH lesions and tumour size conditioning. Example images and labels can be seen in the appendix. We then used different combinations of the real dataset (containing WMH lesions only) and the synthetic one, training models on datasets $R_{R\text{\%}}S_{S\text{\%}}$, where R% is the percentage of real data and S% the percentage of synthetic data. We also train model $R_{WMH}R_{TUM}$ on our real dataset and the BraTS dataset used for training $D_{tum-real-3D}$. Because we do not have ground truth WMH segmentations for the BraTS images, we leave the labels blank.

**Table 11:** Dice score and standard deviation obtained on testing models trained on real data on synthetic test datasets for healthy region segmentation (CSF, GM, WM, DGM and brainstem), tumour (whole tumour and tumour core) and WMH. Note that, because synthetic 2D data cannot be assembled into 3D volumes, the Dice scores for $H_{ID-real-2D}$, $D_{tum-real-2D}$ and $D_{WMH-real-2D}$ were calculated on 2D slices, contrarily to tests on 2D models ran for other experiments.

Healthy					
Model	CSF	GM	WM	DGM	Brainstem
$H_{ID-real-2D}$	$0.942_{0.020}$	$0.940_{0.009}$	$0.953_{0.017}$	$0.819_{0.160}$	$0.793_{0.117}$
$H_{ID-real-3D}$	$0.913_{0.015}$	$0.905_{0.009}$	$0.927_{0.007}$	$0.805_{0.014}$	$0.892_{0.006}$

Tumour			WMH		

Model	Dice (whole)	Dice (core)	Model	Dice (WMH)	

$D_{tum-real-2D}$	$0.663_{0.361}$	$0.358_{0.394}$	$D_{WMH-real-2D}$	$0.383_{0.347}$	
$D_{tum-real-3D}$	$0.677_{0.205}$	$0.527_{0.266}$	$D_{WMH-real-3D}$	$0.632_{0.071}$	

To evaluate the performance of these WMH segmentation models, we test them on two different datasets: the hold-out SABRE dataset used in Section 5.6.3 for WMH, and the hold-out BraTS dataset used in Section 5.6.3 for tumours, BraTS-OTHER (see Table 1). For the first, we compute the Dice score with the ground truth WMH segmentations, but for the second, as we do not have these masks, we compute the number of tumour voxels mislabelled as WMH, normalised by the tumour size, $FP_{tum}$, given by:

(5) $$\frac{1}{\overset{N}{\underset{i=0}{\sum }}I_{TUM-seg,i}==1.0}\overset{N}{\underset{i=0}{\sum }}I_{WMH-seg,i}\cap I_{TUM-seg,i}==1.0$$

where N is the number of voxels, $I_{WMH-seg}$ is the predicted WMH segmentation, and $I_{TUM-seg}$ is the ground truth tumour segmentation.

Results are available in Table 12. We can see that, whereas using only synthetic data leads to a significant decrease in the Dice score obtained on the SABRE WMH test set, the $FP_{tum}$ ratio decreases significantly as we add synthetic data to the real dataset, without compromising the performance on the source SABRE dataset. The best Dice and $FP_{tum}$ results are achieved by $R_{WMH}R_{TUM}$ though, which poses the question of whether synthetic data is really necessary. Nonetheless, when we look at the segmentations themselves on SABRE and BraTS (see Fig. 13), we see that $R_{WMH}R_{TUM}$ does not segment anything for the BraTS dataset, despite the presence of WMH lesions. Because the BraTS WMH masks used for training were blank, the model learnt to map the presence of tumours to the absence of WMH. The rest of the models, however, do segment WMH lesions that are, indeed, present on the BraTS dataset. Note that $R_{0}S_{100}$ performs significantly worse on SABRE than the rest; stressing, as seen in previous experiments, that a gap between real and synthetic domain prevails.

**Table 12:** Performance of segmentation models trained on real, synthetic and hybrid datasets containing WMH lesions and WMH+tumour lesions. Dice is reported for the SABRE WMH hold-out set, and FP_tum_ is reported for the BraTS-OTHER dataset; *: note that R_100_S_0_ corresponds to model D_WMH-real-3D_ from Section 5.6.3.

Training dataset	WMH Dice (on SABRE)	FP_tum_ (on BraTS)
$R_{100}S_{0}$	0.806_0.070_*	0.468_0.220_
$R_{75}S_{25}$	0.802_0.072_*	0.143_0.215_
$R_{50}S_{50}$	0.807_0.073_*	0.141_0.217_
$R_{25}S_{75}$	0.802_0.072_*	0.143_0.216_
$R_{5}S_{95}$	0.810_0.069_	0.025_0.078_
$R_{0}S_{100}$	0.631_0.0100_	0.005_0.017_**
$R_{WMH}R_{TUM}$	0.801_0.071_*	0.000_0.000_*

## 6 Discussion

In this work, we have shown that synthetic labels and images can be used to supplement existing datasets, or serve as surrogate, in a wide range of segmentation tasks. Our approach, which proposes a two-stage disease-conditioned label generator and multi-modal semantic image generator, is able to generate paired datasets in which the user can control which pathologies are present, and which modalities are generated. When used to complement real datasets in segmentation tasks, comprised of both healthy tissues and pathologies, our model improved most of the performance metrics when real data is limited, yielding results that are close to those achieved when using a large amount of real data. Seemingly, when not given any real data, the synthetic datasets can still be used to train models that had fairly good performance on real data, even though it was significantly lower than that achieved by a model trained solely on large real datasets.

### 6.1 2D versus 3D

**Fig. 14:**
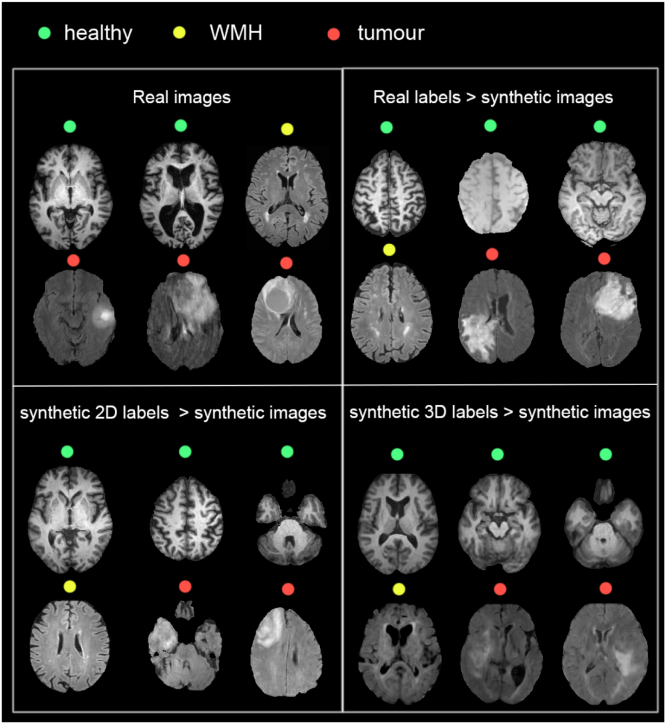
Example healthy and diseased images coming from (top left): real distribution; (top right): images generated from real labels; (bottom left): images generated from synthetic 2D labels; (bottom right): images generated from synthetic 3D labels. T1 was chosen for healthy images to display better tissue contrast, and FLAIR for WMH and tumour images.

**Fig. A.16:**
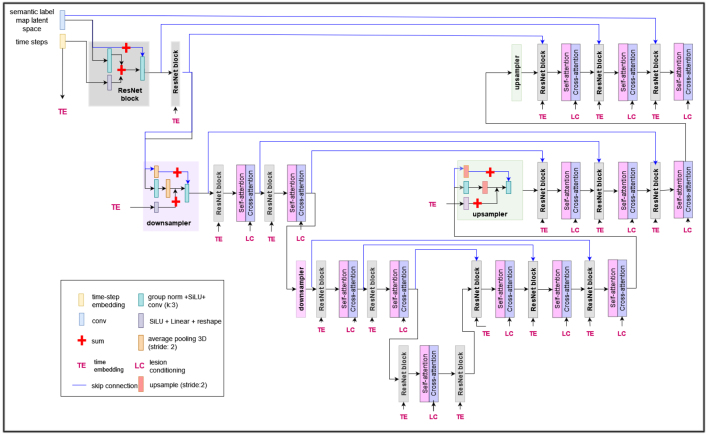
Architecture of the time-gated residual U-Net used to implement the diffusion model.

**Fig. C.17:**
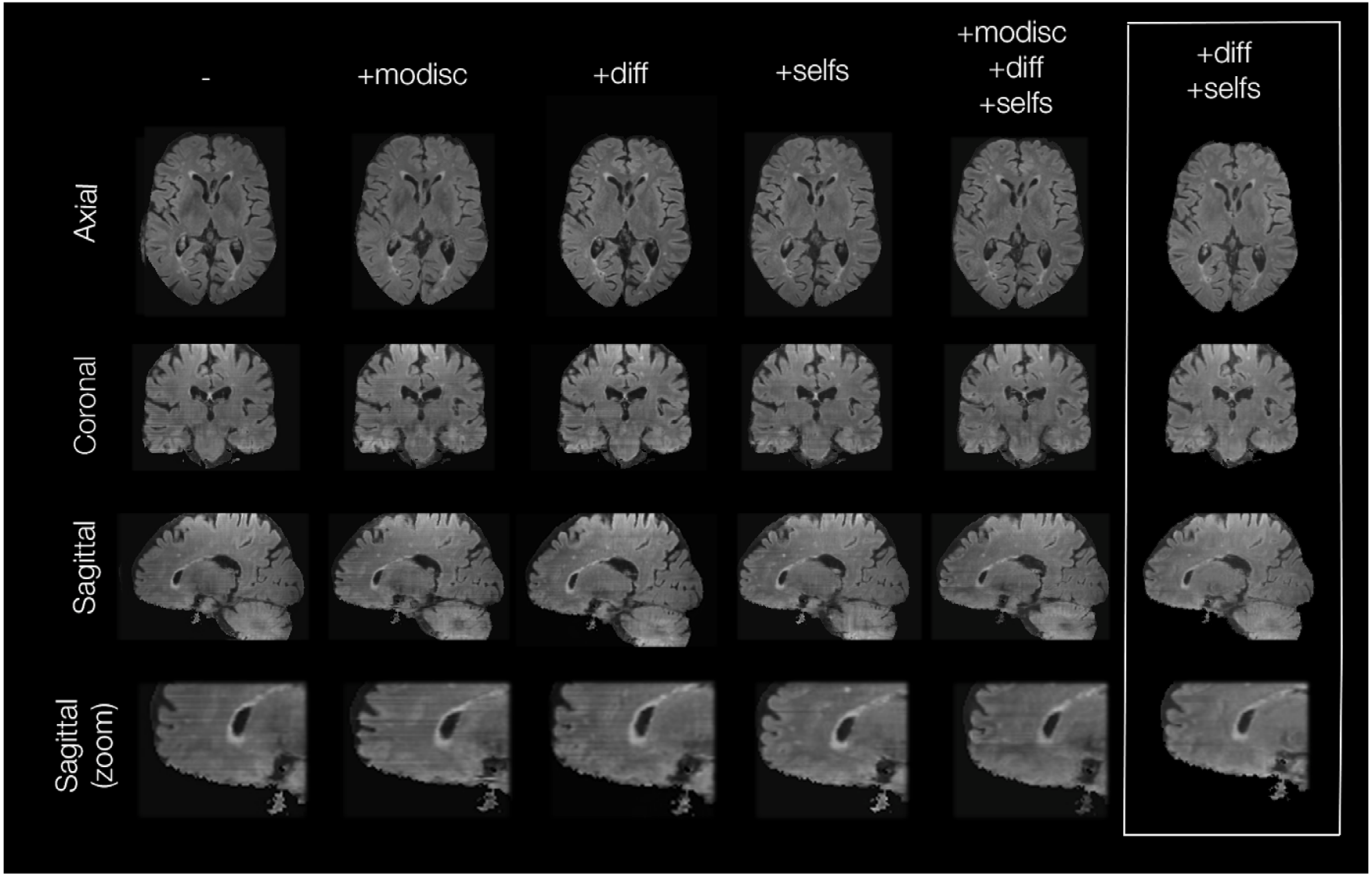
Example reconstructed FLAIR volumes. From top to bottom: axial, coronal, sagittal slices and zoom on the cerebellum of the sagittal slices of the volumes reconstructed from the stack of synthetic T1 slices. From left to right: models using no additional losses (original SPADE network), $L_{mod-disc}$ loss only, unpaired style and semantic slices, $L_{cont}$ loss only, all losses and unpaired style and semantic slices, and the statistically best one, using $L_{cont}$ and unpaired style and semantics slices.

**Fig. C.18:**
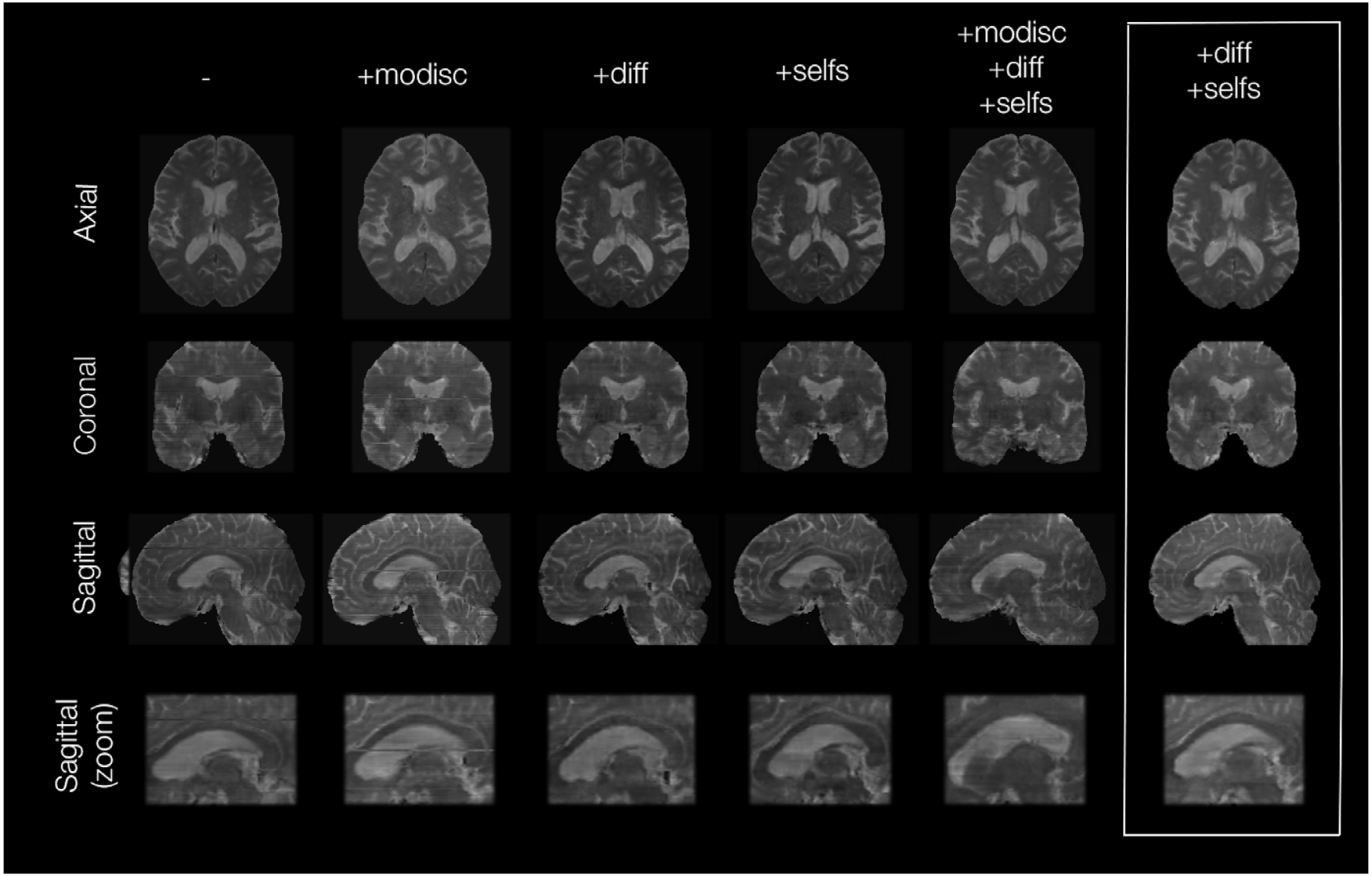
Example reconstructed T2 volumes. From top to bottom: axial, coronal, sagittal slices and zoom on the cerebellum of the sagittal slices of the volumes reconstructed from the stack of synthetic T1 slices. From left to right: models using no additional losses (original SPADE network), $L_{mod-disc}$ loss only, unpaired style and semantic slices, $L_{cont}$ loss only, all losses and unpaired style and semantic slices, and the statistically best one, using $L_{cont}$ and unpaired style and semantics slices.

**Fig. D.19:**
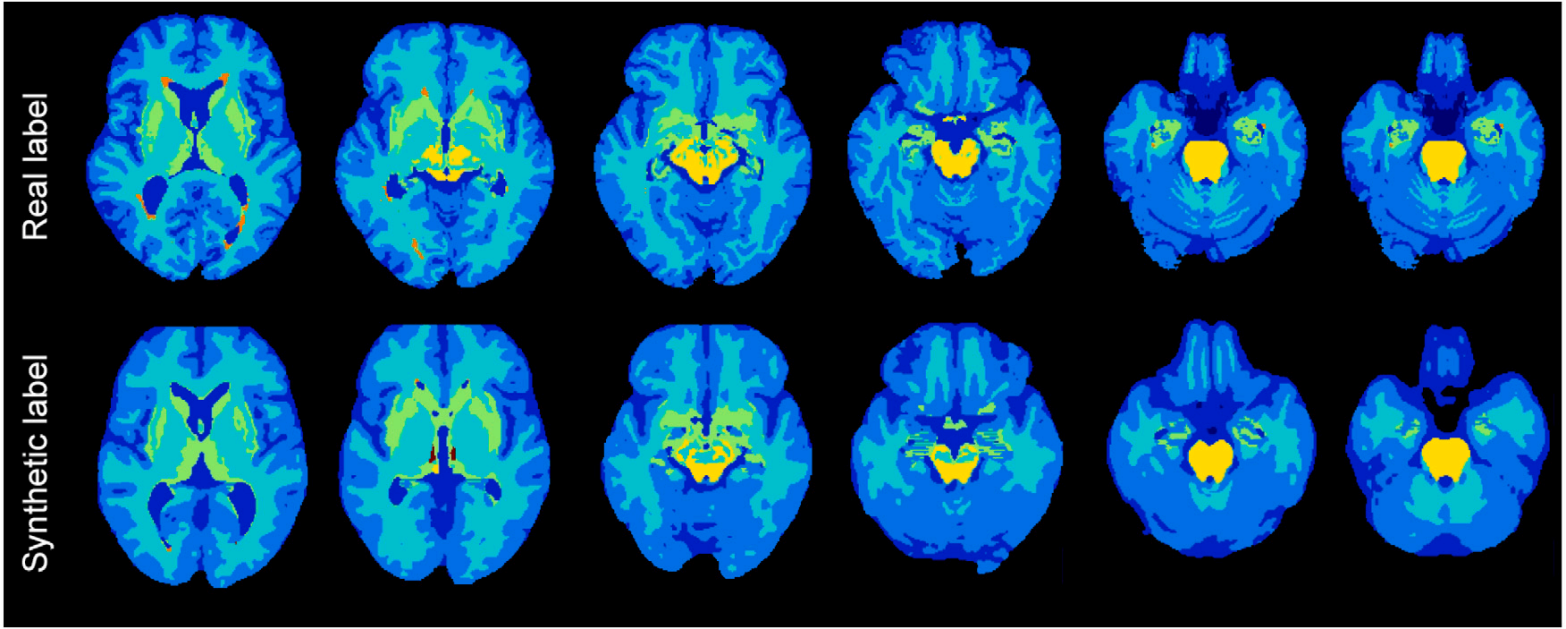
Set of axial slices from the argmaxed probabilistic map of a real (top) and synthetic label (bottom).

**Fig. D.20:**
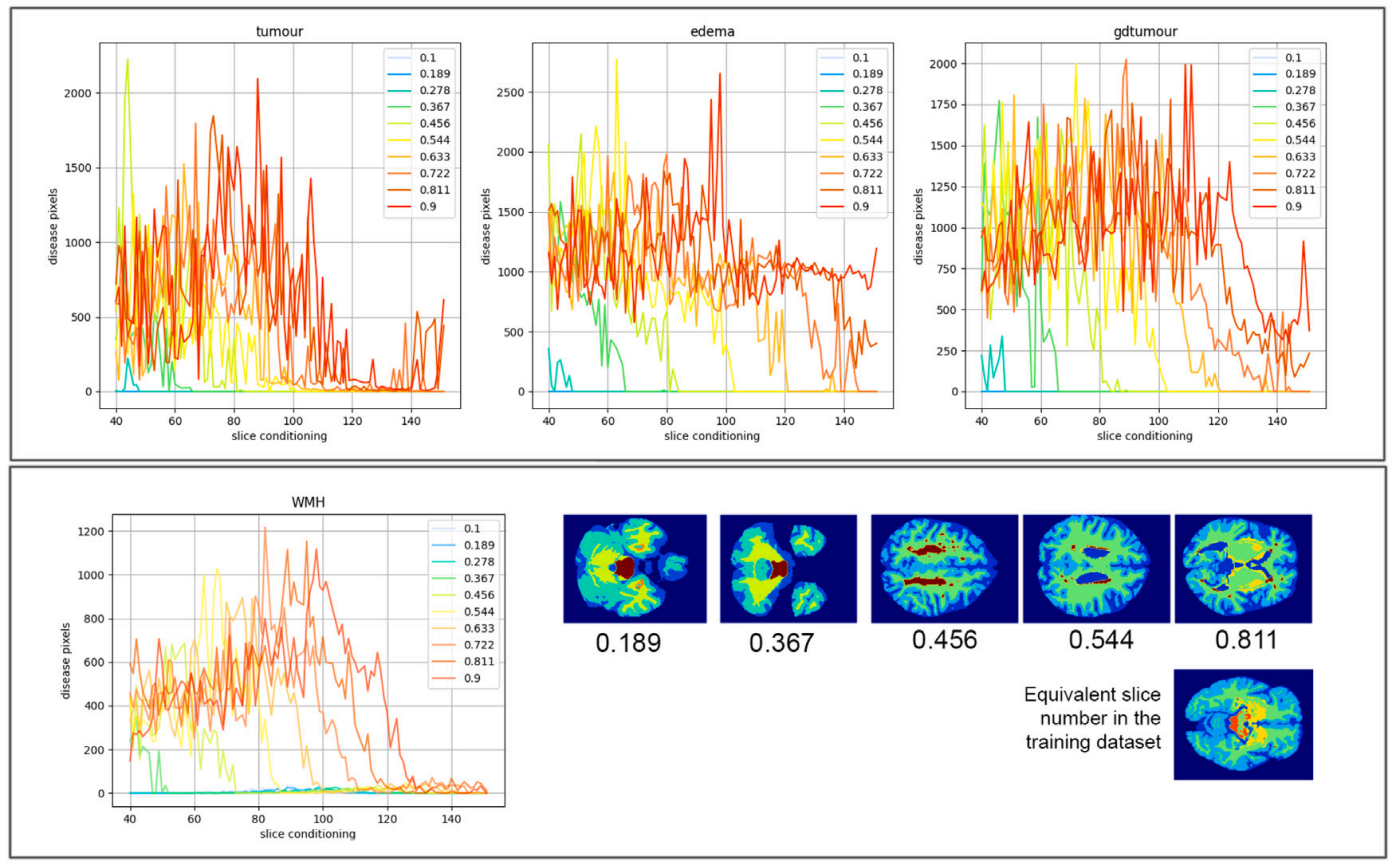
Proportion of lesion pixels as a function of slice conditioning and lesion conditioning values for tumour layers (top) and WMH (bottom). For tumour, each sample had the same value per tumour layer (i.e.: all three, oedema, non-enhancing and GD-enhancing tumour were set to 0.1, 0.189 etc.). On the bottom, example WMH conditioned images for a specific slice number which are displayed along with their WMH conditioning value. Below, an example of that slice number from the training dataset is displayed.

As mentioned earlier, this work is an extension of the work presented in Fernandez et al. (2022) to 3D, where the labels are 3D and the images are reconstructed from generated 2D images. Here, we also enable disease conditioning, which gives the user more control over the subject’s phenotype, resulting in a more useable, versatile model. When we look at the performance of 2D models trained on synthetic 2D data (which was computed on 3D images, by assembling segmented 2D slices), we do not see a lower performance than models trained on synthetic 3D data. Therefore, one could argue whether 3D is really necessary. Other than the existing limitation of assembling 2D slices into a volume, which causes intensity inconsistencies between slices, if we have a look at individual samples generated using synthetic labels, such as those depicted in Fig. 14 or Fig. 12, we see that the visual quality of 2D images generated from 2D labels seems to be better. Indeed, the slices resulting from the 3D labels appear to be less realistic than those produced by the fully 2D pipeline, which can fool a human rater. This can be explained by the availability of more data in 2D generative models (where each 3D image results in more than 200 slices), and by the possibility of having more parameters to model the label distribution. Through the enabling of data augmentation in the label generator training, and the use of techniques to fit the models to GPU, we have tried to push the model capacity and make it as generalisable as possible. Nonetheless, these come with challenges of their own. For example, augmentation can result in unrealistic generated labels, but it is necessary for 3D to avoid overfitting. This evidence of limited capacity is backed up by Fig. 2, where the 3D label generator produced a substantially lower variability across healthy tissues than that seen in real subjects.

However, 3D has advantages of its own. Even though we used conditioning in both 2D and 3D label generators, the 2D model struggled to produce them, as the presence of multiple lesions with different profiles and specific location areas does not make much sense in 2D. While we tried to mitigate this by adding a slice number conditioning, this only worked to a certain extent, with a wide range of conditioning values mapping to seemingly identical locations across the z-axis, and vice-versa. In 3D, on the other hand, conditioning works well, as shown by Fig. 3.

### 6.2 The synthetic-real domain gap

The segmentation experiments, as well as the visual assessment of images, show that, especially for 3D-rendered images, a clear domain gap still exists between synthetic and real domains. If we look at 3D images generated from real labels and compare them to those generated from synthetic labels, we also see a substantial difference (see Fig. 14), showing that this domain gap translates to the semantic domain as well. In addition, the presence of lesions, specifically tumour conditioning, tends to deteriorate the resulting image quality, aggravating the impact of the synthetic labels on the image generator. A reason that might explain this is that BraTS is a heterogeneous dataset with a highly variable quality across the images (some of them having artifacts), and with tumours with very different appearance. This might lead to (1) the overall image generator being more unstable when tumours are present and (2) the image generator “overfitting” certain tumours, causing out-of-domain tumours to have a worse appearance. In addition, the fact that we are treating the different tumour layers as independent conditioning, and that, during sampling, we are randomising the value per layer, might lead to a combination of tumour layers that is too far from the real distribution, and cause the image generator to fail at producing a realistic-looking tumour. Future work should inspect this dependencies to achieve realistic tumour conditioning.

Indeed, it is natural that incorporating heterogeneous datasets and phenotypes deteriorates the image quality, especially in 3D, when the number of hyperparameters is much more limited. Good visual quality has been achieved in previous works in 3D, but they are typically limited to healthy subjects, specific modalities or constrained tasks (Tudosiu et al., 2022, Pinaya et al., 2022, Khader et al., 2023).

One could argue, however, whether it is actually desirable to yield images that are indistinguishable from the originals; as shown by domain randomisation methods (Billot et al., 2023), even unrealistic images can be beneficial for downstream tasks such as segmentation.

### 6.3 Privacy concerns

In Carlini et al. (2023), it is shown that diffusion models are more prone to memorisation than their GAN counterparts. To assess whether labels were being memorised or not, we looked at the closest neighbours of generated labels in the training dataset. We dimed the synthetic labels sufficiently different from their closest real training counterparts to conclude that memorisation is not happening. Nonetheless, we are aware that the criteria to decide whether or not memorisation is occurring, currently, and across the literature, is subjective and non-standardised. In addition to the criteria used, the assessment method (in this case, we used Dice-based similarity) is also a choice of the author(s). Moreover, we saw that, whereas a clear difference exists in terms of healthy brain anatomy, synthetic tumours share more similarities with samples from the training dataset. This is discussed in Carlini et al. (2023): tumours, although widely present in our training set, have a more unique shape and location, and are closer to the concept of *outlier* and, therefore, more prone to be memorised. As we add pathological data to make our model more generalisable, we wonder whether our model will be able to not fall under data memorisation.

As argued in the experimental section, disentangling the semantic and contrast generation processes can help avoid memorisation, especially if different subsets of data are used to train each component. We showed, in Section 5.5, that the image encoder does not seem to be memorising the training distribution. However, entirely out-of-distribution data seems to fall within the same K-Means cluster, showcasing a different behaviour. This, nonetheless, still does not allow separate distributions. Since, nevertheless, we limited this experiment to healthy or WMH data, further work should extend this experiment to outliers.

### 6.4 Conclusion and further work

In this work, we propose a generative model for labels and multi-modal images, where the user has control over the lesions present and the desired contrasts. Our model is capable of providing privacy-preserving datasets that can be used in a wide range of segmentation tasks, either supplementing small, real datasets or replacing them if the end-user does not have access to any real paired image. This approach can, therefore, tackle data scarcity in medical imaging, easing the translation of segmentation models towards unseen modalities or phenotypes.

## Code availability

The code for this project has been released on Github (https://github.com/virginiafdez/brainSPADE23D.git). Additionally to this repository, the MONAI Generative Models package must be installed from https://github.com/Project-MONAI/GenerativeModels.git.

## CRediT authorship contribution statement

**Virginia Fernandez:** Writing – original draft, Visualization, Validation, Software, Methodology, Investigation, Conceptualization. **Walter Hugo Lopez Pinaya:** Software, Methodology, Data curation. **Pedro Borges:** Writing – review & editing, Methodology, Data curation. **Mark S. Graham:** Writing – review & editing, Software, Data curation. **Petru-Daniel Tudosiu:** Software. **Tom Vercauteren:** Writing – review & editing, Supervision. **M. Jorge Cardoso:** Writing – review & editing, Supervision, Resources, Project administration, Data curation, Conceptualization.

## Declaration of competing interest

The authors declare the following financial interests/personal relationships which may be considered as potential competing interests: Virginia Fernandez reports financial support, administrative support, and travel were provided by EPSRC Centre for Doctoral Training in Smart Medical Imaging. Tom Vercauteren reports a relationship with Hypervision Surgical that includes: board membership and equity or stocks. If there are other authors, they declare that they have no known competing financial interests or personal relationships that could have appeared to influence the work reported in this paper.

## Figures and Tables

**Fig. A.15:**
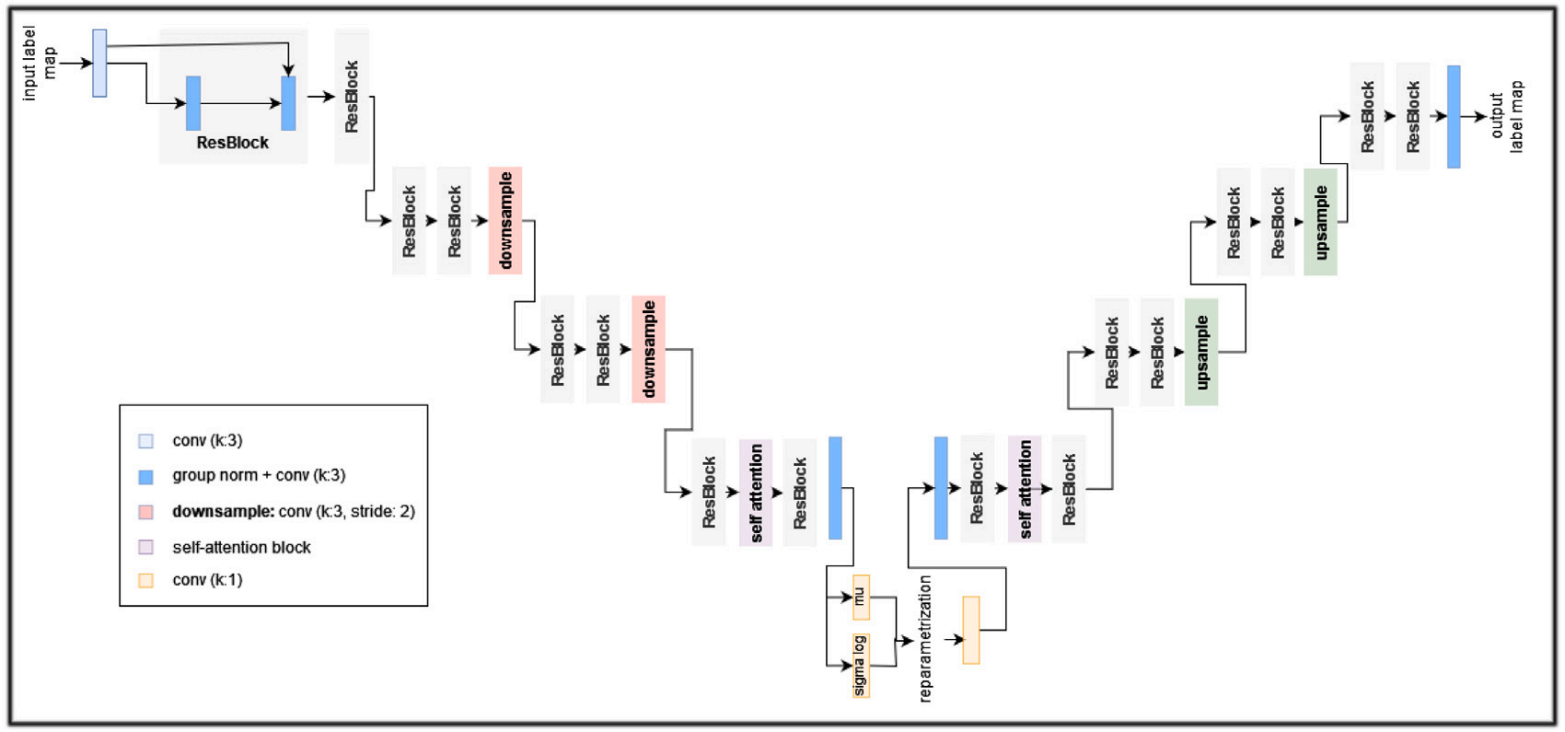
Architecture of the spatial VAE used to encode–decode the semantic label maps and bring them to a latent space.

## Data Availability

Datasets used in this work are either private and shareable upon request by the owners (references in the paper) or publicly available in the cited sources. The code is on Github (link on paper).

## Appendix A Architectures

The architecture for the spatial VAE and the diffusion model used for the label generator stage are depicted in Fig. A.15 (S-VAE) and Fig. A.16 (DM). The architecture of the image generator has been implemented from and is depicted in Park et al. (2019).

## Appendix B Implementation details

### B.1 Obtention of 2D datasets for training

To train the 2D label generator and the image generator, we took slices from the original 3D volumes. These slices were sampled to ensure an equal balance between disease and non-diseased slices (we consider a slice as ‘diseased’ if it has at least 5 pixels associated with white matter hyperintensities (WMH) or any tumour sub-category). On the other hand, since uppermost and lowermost brain slices contain mostly non-brain tissue, which is not relevant for our downstream segmentation tasks, we calculated the ratio between brain and total voxels for each slice and discarded the ones with a percentage lower than 10%. Before discarding these slices, we associated the slice index z to the sliced images. This number was then divided by 256 (the total number of axial slices in the MNI space) to obtain the relative slice number used to condition the 2D label generator.

### B.2 Label generator

The weights used for the 2D and the 3D label generator are the following:

– $\lambda_{perc}$= 0.002 (2D) and 0.001 (3D)
– $\lambda_{adv}$= 0.005 (2D) and 0
– $\lambda_{fl}$= 1.0 (2D) and 1.0 (3D)
– $\lambda_{kld}$= 0.000001 (2D) and 0.0 (2D) and 0.5 (3D)
– $\lambda_{nr}$= 0.0 (2D) and 0.5 (3D)

Adam optimiser with $\beta_{1}=0.99$ and $\beta_{2}=0.999$ was used. For the S-VAE, we used a learning rate of $5\cdot 10^{-5}$ for the generator (2D and 3D) and of 10^−4^ for the discriminator (2D). In the 2D case, an exponential decay with γ=0.999995 was applied. For the LDMs, a learning rate of $2.5\cdot 10^{-4}$ was used. The variance schedule was linear, starting at 0.0015 and ending at 0.0195.

The 2D and 3D S-VAEs were trained for 200 epochs, and the 2D and 3D DMs were trained for 130 and 275 epochs respectively, on an Nvidia DGX-A100 GPU.

### B.3 Image generator

The weights used for the image generator were tuned empirically, and are the following:

– $\lambda_{adv}$ (adversarial loss) = 1.0
– $\lambda_{kld}$ (KLD loss) = 0.0005
– $\lambda_{feat}$ (feature-matching loss) = 0.25
– $\lambda_{perc}$ (perceptual loss) = 1.5
– $\lambda_{contrastive}$ (contrastive loss on the latent space) = 1.0
– $\lambda_{modisc-mod}$ (modality-discriminator loss) = 0.75
– $\lambda_{modisc-dat}$ (dataset-discriminator loss) = 0.3

Adam optimiser with $\beta_{1}=0.5$ and $\beta_{2}=0.99$ was used. We trained the models on an Nvidia RTX8000 GPU for 200 epochs with a fixed learning rate of $2\cdot 10^{-4}$ and then applied a linear decay for 50 more epochs. The training dataset size consisted of 18 000 slices.

### B.4 Modality and dataset discriminator network

To train the modality and dataset classifier used to yield the *modisc* loss used for the image generator, we used a Densenet121 architectures with two parallel output layers, one to classify the modality (T1, FLAIR or T2) and the other one to classify the dataset (SABRE, BraTs or ADNI). These modalities and datasets were one-hot encoded. Binary-cross-entropy with logits (BCE with logits) was used to train this network, summing the BCE for each of the tasks, using Adam optimizer and an exponential decaying learning rate going from 0.01 to 0.0001 across 50 epochs. The data loading pipeline was equal to that used for the main image generator training. The final accuracy for modalities was 91.61% (AUC 0.986) and that for datasets was 78.33% (AUC 0.823). The network weights were frozen for the *modisc* loss calculation.

## Appendix C Ablation study

The ablation study consisted of training models on the following loss functions:

1. Original SPADE losses
2. Original SPADE losses + unpaired label and image slices
3. Original SPADE losses + modality and classifier discriminator loss
4. Original SPADE losses + contrastive loss
5. Original SPADE losses + unpaired label and image slices + modality and classifier discriminator loss + contrastive loss

Because we aim for an increased match between the contrasts of generated neighbouring slices, we evaluated the performance of these models on 12 3D volumes, reconstructed by forward-passing the segmentation slices through them. For each volume, we fixed the input style image to the central slice of the image volume. We calculated the SSIM between neighbouring slices for all the slice pairs and volumes, and normalised them by the SSIM between neighbouring slices in the ground truth volumes. We assessed the reconstructed volumes visually, and we performed a statistical analysis of the SSIM values using paired one-sided T-tests with Bonferroni correction.

The paired statistical one-sided t-tests revealed that model 4. yields higher significant SSIM ratio than the others (p-value < 0.05 for all pairs). Model 2. is also significantly better than 5., 3. and 1. (p-value < 0.05) for all pairs. No statistical improvement is shown for the model using all the losses.

Visual examples for FLAIR and T2 contrasts can be seen in supplementary Fig. C.17, Fig. C.18.

## Appendix D Quality of the generated labels

**Tissue proportions:** To get an intuition as to why we observed some major discrepancies between the average number of voxels per tissue in the labels sampled from the 3D label generator, compared to real labels, we drew some images and compared them side by side. Fig. D.19 shows a real and synthetic label.

**Conditioning in 2D:** As explained in the main results, we could not quantify or observe the effect of increasing the lesion conditioning in 2D as well as we did in 2D, due to the interplay between the slice and lesion conditionings. Fig. D.20 shows the lesion proportion per lesion and slice conditioning value. Whereas the tumour plots are rather noisy, we can see a pattern in WMH where each lesion value plot has a peak at a specific slice, with that slice number increasing as the lesion value increases. This points towards the idea that the lesion conditioning only works when the combination of slice and WMH conditioning matches that observed in the training dataset. For tumours, we do not see these patterns because they do not have an approximate constant appearance per slice as do WMH: their location and size varies across subjects. If we look at the visual examples, we clearly see that the slice conditioning, which worked in a healthy setting, as shown by Fig. 2, does not work for lesion conditioning values that are unlikely to happen. Overall, this proves that lesion extrapolation does not work in 2D. If we were to use a lesion and slice conditioned-model, these two elements should be disentangled to ensure that they work independently to achieve good results.

## Footnotes

1 Note that, even though the resulting datasets are 3D, the image generator employs a 2D model.

2 The ADNI was launched in 2003 as a public–private partnership, led by Principal Investigator Michael W. Weiner, MD. The primary goal of ADNI has been to test whether serial MRI, positron emission tomography (PET), other biological markers, and clinical and neuropsychological assessment can be combined to measure the progression of mild cognitive impairment (MCI) and early Alzheimer’s disease (AD). For up-to-date information, see www.adni-info.org.
